# TELS: A Novel Computational Framework for Identifying Motif Signatures of Transcribed Enhancers

**DOI:** 10.1016/j.gpb.2018.05.003

**Published:** 2018-12-19

**Authors:** Dimitrios Kleftogiannis, Haitham Ashoor, Vladimir B. Bajic

**Affiliations:** 1 Centre for Evolution and Cancer, Division of Molecular Pathology, The Institute of Cancer Research (ICR), London SM2 5NG, United Kingdom; 2 The Jackson Laboratory for Genomic Medicine, Farmington, CT 06032, USA; 3 Computational Bioscience Research Center (CBRC), Computer, Electrical and Mathematical Sciences and Engineering Division (CEMSE), King Abdullah University of Science and Technology (KAUST), Thuwal 23955-6900, Saudi Arabia

**Keywords:** Sequence analysis, Machine learning, Transcription regulation, Transcribed enhancer, Motif identification

## Abstract

In mammalian cells, **transcribed enhancers** (TrEns) play important roles in the initiation of gene expression and maintenance of gene expression levels in a spatiotemporal manner. One of the most challenging questions is how the genomic characteristics of enhancers relate to enhancer activities. To date, only a limited number of enhancer sequence characteristics have been investigated, leaving space for exploring the enhancers’ DNA code in a more systematic way. To address this problem, we developed a novel computational framework, Transcribed Enhancer Landscape Search (TELS), aimed at identifying predictive cell type/tissue-specific motif signatures of TrEns. As a case study, we used TELS to compile a comprehensive catalog of motif signatures for all known TrEns identified by the FANTOM5 consortium across 112 human primary cells and tissues. Our results confirm that combinations of different short motifs characterize in an optimized manner cell type/tissue-specific TrEns. Our study is the first to report combinations of motifs that maximize classification performance of TrEns exclusively transcribed in one cell type/tissue from TrEns exclusively transcribed in different cell types/tissues. Moreover, we also report 31 motif signatures predictive of enhancers’ broad activity. TELS codes and material are publicly available at http://www.cbrc.kaust.edu.sa/TELS.

## Introduction

In mammalian cells, spatial and temporal activation of gene transcription and maintenance of expression levels is coordinated (mainly) by interactions between DNA regulatory elements, the most prominent being promoters and enhancers [1]. Promoters surround the transcription start sites (TSSs) of genes and represent the class of proximal regulatory elements. Specific regions in promoters are used as binding sites responsible for recruiting and anchoring the transcriptional machinery [2]. On the other hand, enhancers, frequently called distal regulatory elements, are positioned a few thousands or many thousands of base pairs (bp) downstream or upstream of the TSSs of genes. Typically, enhancers activate their target genes via physical interactions with transcription factors (TFs), as well as co-activators, and/or via chromatin remodeling processes [3], [4]. Results obtained from the cap analysis of gene expression (CAGE) show that transcription in enhancers mediated by RNA polymerase II (RNAPII) occurs on a genome-wide scale [5]. Enhancers’ transcription produces enhancer-derived RNAs (eRNAs), a class of non-coding RNAs whose functions are unclear [6], [7]. It is interesting to note that it may be difficult to clearly separate enhancers from promoters, based on the transcriptional activation similarity, since both categories of DNA regulatory regions act as promoters but generate different classes of transcripts [8], [9], [10].

Several enhancer identification methods, covering both experimental and computational approaches, have been subject of review articles [11], [12]. Using the available enhancer-related information [13], [14], a number of studies linked variations in enhancer sequences to disease phenotypes, and development/progression of cancer [15], [16], [17], [18], [19]. Thus, deciphering the genomic characteristics of enhancers may help to understand better enhancers’ functional roles.

Up to now, there are several approaches to analyze enhancers’ DNA characteristics and associate sequence properties to enhancer activities [20], [21], [22]. However, only a limited number of cases, in terms of studied enhancer sequences, sequence motifs (*e.g.*, kmers of length 6–8 bp), tissues, and organisms (*e.g.*, mice or *Drosophila*), have previously been examined or validated experimentally [23] (*i.e.*, by massive parallel reporter assays; MPRAs), leaving space for further investigations.

With all of the aforementioned issues in mind, we present the Transcribed Enhancer Landscape Search (TELS), a novel bioinformatics framework that applies logistic regression (LR) coupled with a dimensionality reduction algorithm, aimed at identifying systematically the most informative combinations of short sequence motifs of TrEns in the human genome. As a case study, we applied TELS to the atlas of CAGE-defined TrEns that covers 112 human primary cells and tissues [5].

As importantly, TELS contributes (1) comprehensive exploration of the genomic landscape of human TrEns using all available experimentally-verified enhancers by the FANTOM5 consortium; (2) identification of novel combinations of short sequence motifs (equally denoted as DNA signatures or motif signatures) in TrEn sequences that are characteristic and predictive of TrEns in a cell type/tissue-specific manner; (3) the identified motifs allowing for more accurate discrimination of TrEns compared to motif sets reported by other studies; and (4) the identified motifs performing equally well on the category of chromatin-defined enhancers identified by the Encyclopedia of DNA Elements (ENCODE) consortium.

We report for the first time the combinations of short motifs that discriminate successfully FANTOM5 enhancers expressed and transcribed exclusively in a cell type/tissue-specific manner from enhancers expressed and transcribed exclusively in multiple primary cells or tissues. Our results demonstrate that the proposed framework leads to the discovery of informative motif signatures of TrEn sequences. Thus, it opens possibilities for analyzing systematically the genomic landscape of human TrEns and it can serve as a paradigm for similar studies in other mammals.

## Methods

### Data availability

The primary datasets included in this study are derived from the FANTOM5 atlas of TrEns [5]. Using a large number of primary cells and tissues, Andersson et al. identified bi-directional TrEns via CAGE experiments. All enhancer samples were obtained from the atlas webpage (http://enhancer.binf.ku.dk/presets/) accessed in November 2016. Details about the TrEn identification pipeline from CAGE and other information about the primary data have been described previously [5].

For further validation of our findings, we use the list of ‘strong’ enhancers reported by the ENCODE integrative annotation [24]. Details about the ‘strong’ enhancer identification process have been described previously [24]. From the cell-line-specific lists of ‘strong’ enhancers, we consider only the sequences that do not overlap with CAGE-defined enhancers from the FANTOM5 TrEn atlas [5]. This guarantees that the ENCODE data we used for testing (*i.e.*, positive class) are different from the FANTOM5 data that we used for training the models and identifying motif signatures.

All TELS source codes for reproducing the results are publicly available at http://www.cbrc.kaust.edu.sa/TELS/ under an Educational Community Licence (ECL-2.0).

### Definition of positive and negative datasets for motif selection

To identify motif signatures of TrEns, we used the following three datasets that are considered ‘positive’ data for training, including ‘all facets’ enhancers (http://enhancer.binf.ku.dk/presets/facet_expressed_enhancers.tgz), the ‘robust set’ enhancers (http://enhancer.binf.ku.dk/presets/robust_enhancers.bed), and the ‘exclusively transcribed’ enhancers. (1) The dataset for ‘all facets’ enhancers contains enhancers transcribed in all FANTOM5 facets from 112 cell types and tissues (*i.e.*, 112 TrEn sets), covering 197,373 genomic sequences including duplicates since some TrEns are expressed and transcribed in more than one cell type/tissue. (2) The dataset for the ‘robust set’ enhancers contains the enhancers transcribed at a significant expression level in at least one FANTOM5 primary cell/tissue, covering 38,554 genomic sequences. (3) We denoted as exclusively transcribed enhancers those TrEns that are transcribed in only one FANTOM5 cell type/tissue. We generate a list of exclusively transcribed enhancers for every cell type/tissue from the ‘all facets’ dataset described above. Basophil and granulocyte cell types have no exclusively transcribed enhancers based on the data we used. We also exclude from ‘exclusively transcribed’ enhancer dataset cell types/tissues with less than five exclusively transcribed enhancers. This results in 96 out of 112 potential datasets (one set per cell type/tissue).

Generating ‘negative’ data for the previously described ‘positive’ datasets without experimental validation (*i.e.*, MPRA or STARR-seq) is a challenging task, since it is unclear how to infer computationally whether or not a particular DNA sequence has enhancer activity in a cell type or tissue. Thus, in the absence of a ‘gold-standard’ negative baseline set, any attempt for generating negative control dataset can be criticized as not being optimal. To mitigate this problem, we considered two alternative approaches: (1) based on synthetically generated sequences; and (2) using TrEns expressed in cell types/tissues different than the one of interest.

The rationale behind approach (1) derives from studies showing that mutations in enhancer sequences disrupt the enhancer’s activity [12], [16], [18], [19], [23]. Thus, for every TrEn in the ‘all facets’ dataset as well as for the ‘robust set’, we introduce ‘noise’ by mutating randomly every TrEn sequence. Utilization of negative data ‘corrupted’ by synthetically generated noise (in our case it is a random combination of single nucleotide substitutions), is a common practice in machine learning with many applications in image recognition [25]. This approach gives us a more generic representation of the non-enhancer class, as our aim is to capture properties of enhancers that can differentiate enhancers from other biological sequences with non-enhancer activity. Due to the random nature of mutations introduced during the negative dataset generation process, our derived results may not be optimal but optimized, as we do not know what the ‘best’ negative dataset for this problem is. We generate in total 197,373 negative controls across 112 cell types/tissues named as ‘all facets random controls’ and 38,554 negative controls named as ‘robust set random control’, respectively. Note that throughout the previous data generation process, we make sure that none of the randomly generated sequences belongs to the superset of TrEns identified by FANTOM5. For approach (2), we follow the ‘one *vs.* all’ paradigm and for every cell type/tissue-specific ‘exclusively transcribed’ enhancer set, we generate a negative set that contains exclusively transcribed enhancers from all other cell types/tissues but not from the one of interest. This process resulted in 96 negative sets and such dataset is denoted as ‘negative exclusively transcribed’.

### DNA sequence encoding

To encode the input datasets for further use by TELS, we transform all ‘positive’ and ‘negative’ data samples into numerical vectors. In TELS, we focus on small sequence motifs. In this way, we consider the intrinsic DNA properties of TrEns and we complement similar studies that focus on known motifs usually of length of 6 or 10 bp. We also note that TELS does not require prior knowledge of TF binding sites (TFBSs) based on ChIP-seq or other type of input information.

The deployed vectors contain 346 variables (equally denoted in the current study as sequence motifs, motifs, or features) that describe the enhancers’ genomic specificity. These variables are grouped into five categories: (1) four single nucleotide frequencies; (2) six aggregate frequencies of two nucleotides (*e.g.*, A + C); (3) 16 dinucleotide frequencies; (4) 64 trinucleotide frequencies; and (5) 256 tetranucleotide frequencies. To avoid any bias introduced by the length of the sequences, we normalize all values of the vectors by the sequence length.

### TELS implementation

**Figure 1:**
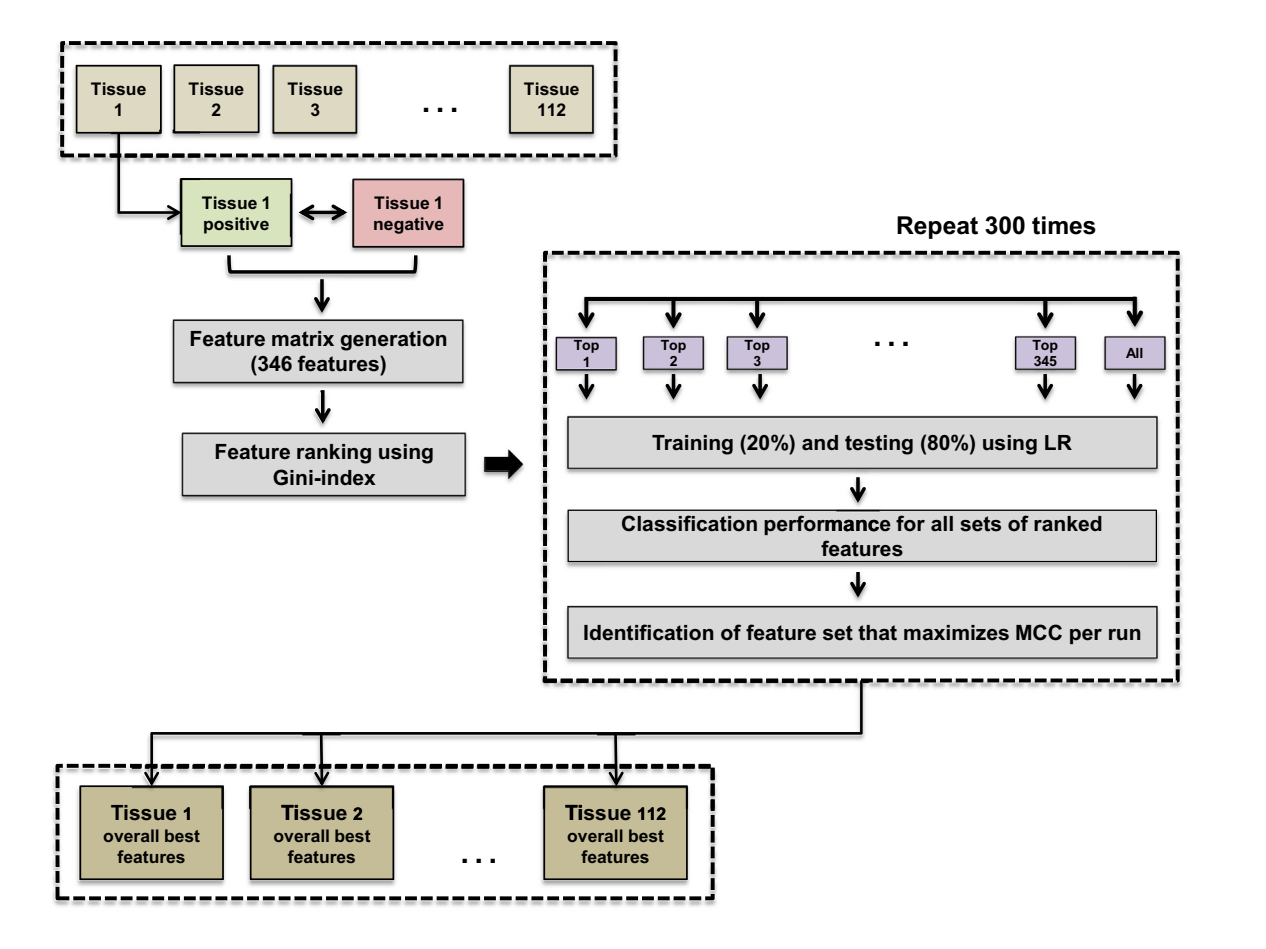
**Schematic diagram of the proposed computational framework** The important phases of TELS are summarized, which include feature matrix generation, feature ranking using Gini-index, training and testing using LR, performance classification, as well as identification of feature sets that maximize MCC per run. LR, logistic regression; MCC, Matthews correlation coefficient.

TELS works in two phases. In phase 1, TELS identifies candidate combinations of sequence motifs that characterize the class of interest. In phase 2, for every candidate combination of motifs, TELS assesses its significance by measuring the classification performance for discriminating ‘positive’ from ‘negative’ data. A simple flowchart of the developed pipeline is presented in Figure 1. The objective of TELS is to select the combination of motifs that maximizes separation between ‘positive’ and ‘negative’ data. Typically, determining the relative importance of a set of predictor variables via computational techniques may be used to associate differences between the considered data classes. Such information can be further utilized to identify sequence characteristics that are predictive of TrEn cell type/tissue-specific activities.

#### Phase 1: Feature selection

To identify candidate combinations of motifs, TELS uses filtering feature selection (FS) techniques. The FS problem in bioinformatics is very well studied [26], [27], [28], [29], [30], [31], [32], [33], [34] and it is well documented that FS is a strongly ‘data-dependent’ process. Among the proposed FS methods, heuristic approaches have the advantage of being able to exploit more combinations of features. However, heuristic approaches (*e.g.*, based on genetic algorithms) introduce higher algorithmic complexity and exponential computational cost in contrast to filtering methods that run fast and thus heuristic approaches are more suitable for problems in higher dimensions. TELS first ranks the 346 individual variables using the Gini-index based FS. We decided to use Gini-index after comparison with two other state-of-the-art algorithms for FS, namely minimum redundancy maximum relevance criterion (mRMR) [35] and Fisher’s test-based FS [34] (Figure S1). We used the Gini-index implementation from the feature selection toolbox (FEAST) in Matlab R2014b. More details about Gini-index FS can be found in the subsection named ‘Gini-based feature selection’ (File S1). As importantly, features ranked by filtering methods are considered ‘independently’, which may lead to suboptimal classification performance. In other words, from a pool of 346 ranked variables based on their significance assessed by the Gini-index, it is not clear which combination characterizes in an optimized manner the class of interest. To mitigate this problem, we applied in phase 2 a greedy approach and assessed the significance of different sets of the ranked features (starting with the top 1, top 2, top 3 and up to top K, where K is 346), which is the total number of variables we used by measuring the classification performance of every candidate combination.

#### Phase 2: Classification

The objective of the classification step is to select the combination of motifs that minimizes the classification error based on the Matthews correlation coefficient (MCC). For this task, TELS utilizes the LR classifier. LR is a simple linear classification method, which runs fast and avoids extensive optimization of model parameters that frequently leads to poor performance on unseen data [36]. The implementation is made in Matlab R2014b using built-in functions for LR (‘glmfit’ function with the default setting without regularization). In one classification run with LR and one candidate motif set, we randomly split the ‘positive’ and ‘negative’ data into testing and training sets. We use 20% of the total size of ‘positive’ and 20% of the total size of ‘negative’ samples for training, whereas the remaining 80% from each set is kept for testing. We decided to use a much smaller fraction (*i.e.*, 20%) of the available data for a training to achieve better generalization capabilities in unseen cases. To account for the potential biases introduced by the selection of negative data, we repeat the training process for 300 runs when for each run the aforementioned random split of data is performed. Consequently, each candidate combination of top-ranked motif sets (*i.e.*, 346), is evaluated 300 times, and characterized by the average classification performance of multiple independent runs. This way guarantees an equitable selection of combinations of motifs that maximizes classification performance. We consider the geometric mean of sensitivity and specificity (GM), positive predictive value (PPV), MCC, area under receiver operating characteristic curve (AUROC), and area under precision–recall curve (AUPRC) as representative classification performance metrics. All performance metric formulas can be found in the subsection named ‘Classification performance metrics (File S1)’.

## Results and discussion

### Analyzing all FANTOM5 cell types and tissues

In this subsection we focus on the results of the analysis of FANTOM5 TrEns from all available cell types/tissues, aimed to compile an atlas of motif sets that discriminate effectively TrEns. To do this, we analysed the FANTOM5 dataset called ‘all facets’ and the negative control dataset called ‘all facets random controls’ using TELS. Our analysis shows that the combinations of motifs identified using TELS discriminate effectively TrEns across 112 cell types/tissues, with an average classification performance of 85.94% for PPV, 86.06% for GM (Figure S2), 0.934 for AUROC, and 0.926 for AUPRC. Figure S3 provides all AUROC and AUPRC values for the cell types/tissues included in the dataset. In Figure S4, we show as an example ROC and PRC for 49 out of 112 cell types/tissues from FANTOM5. The remaining ROC and PR curves are available online in our web repository (http://www.cbrc.kaust.edu.sa/TELS/).

**Figure 2:**
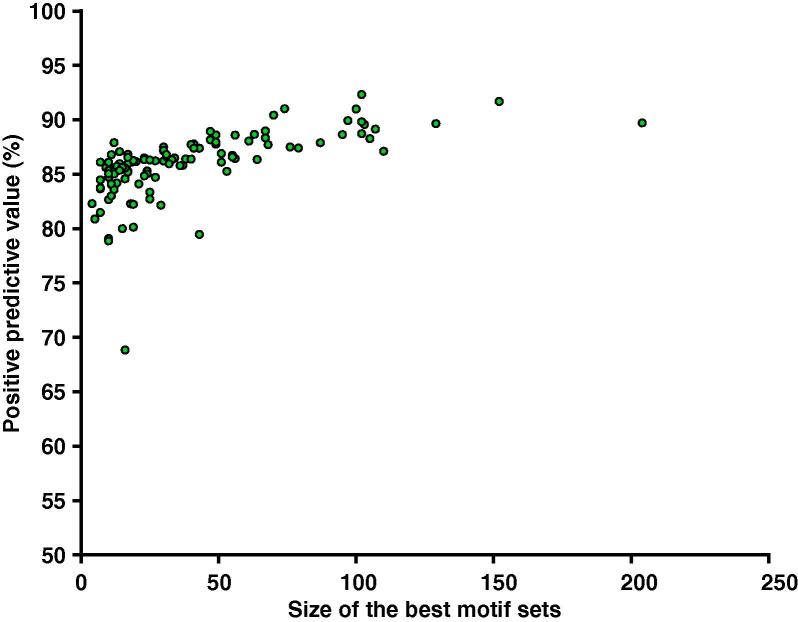
**Classification performance of TELS on the FANTOM5 ‘all-facets’ dataset** The figure shows the corresponding number of motifs that maximizes MCC (*i.e.*, called overall ‘best’ motifs) selected by TELS per cell type and tissue (X axis) versus the positive predictive value achieved using the corresponding motif set (Y axis) across 112 cell types/tissues from the FANTOM5 ‘all-facets’ dataset.

Figure 2 shows the number of selected motifs ranging from 204 to 4, which correspond to the maximum and minimum numbers of motifs that discriminate efficiently cell/tissue-specific TrEns from the random control data, a proxy of non-enhancer activity. At a threshold of 80% of PPV, we observed that the identified motif signatures classify cell type/tissue-specific TrEns with high accuracy in 95% of cases (107 out of 112 cell types/tissues). This suggests that the identified combinations of sequence motifs capture a great portion of the sequence specificities required in TrEns. Figure S5 shows the detailed atlas of identified motifs across 112 cell types/tissues. It is evident that the identified combinations of motifs do not overlap significantly across different cell types/tissues. However, some motifs are almost always selected across the available cell types/tissues.

To investigate further the identified sets of motif signatures, in the supplementary subsection named ‘Analysis of motif signatures across tissues that belong to different developmental stages’ (File S1), we provide a case study using nine randomly selected tissues that belong to three different developmental stages, according to the Embryonic Development & Stem Cell Compendium (https://discovery.lifemapsc.com/in-vivo-development), namely ectoderm (brain, spinal cord, and eye), mesoderm (kidney, heart, and spleen), and endoderm (lung, liver, and pancreas). Figure S6 presents the similarity of informative motifs (Figure S6A) and TrEns (Figure S6B) sequences across different developmental stages.

All observations from Figures 2, S4–S6 suggest that TrEns display cell type/tissue specific motif signatures that are successfully identified by TELS. Overall, these results support the hypothesis that specific genomic characteristics enable TrEns to operate in a highly cell type/tissue-specific manner and for this reason the identified motifs vary across different cell types/tissues.

### Analyzing TrEns expressed in at least one FANTOM5 cell type or tissue

In this subsection we focus on the analysis of TrEns expressed in at least one FANTOM5 cell type or tissue. Our goal is to identify motif signatures that allows us to discriminate the FANTOM5 ‘robust set’ TrEns from the ‘robust set of random control’ dataset, with maximized classification performance. Our results across 300 experiments show that the motif sets identified by TELS are able to identify TrEns from the ‘robust set’ with an average PPV and GM of 79.70% and 80.47%, respectively.

**Figure 3:**
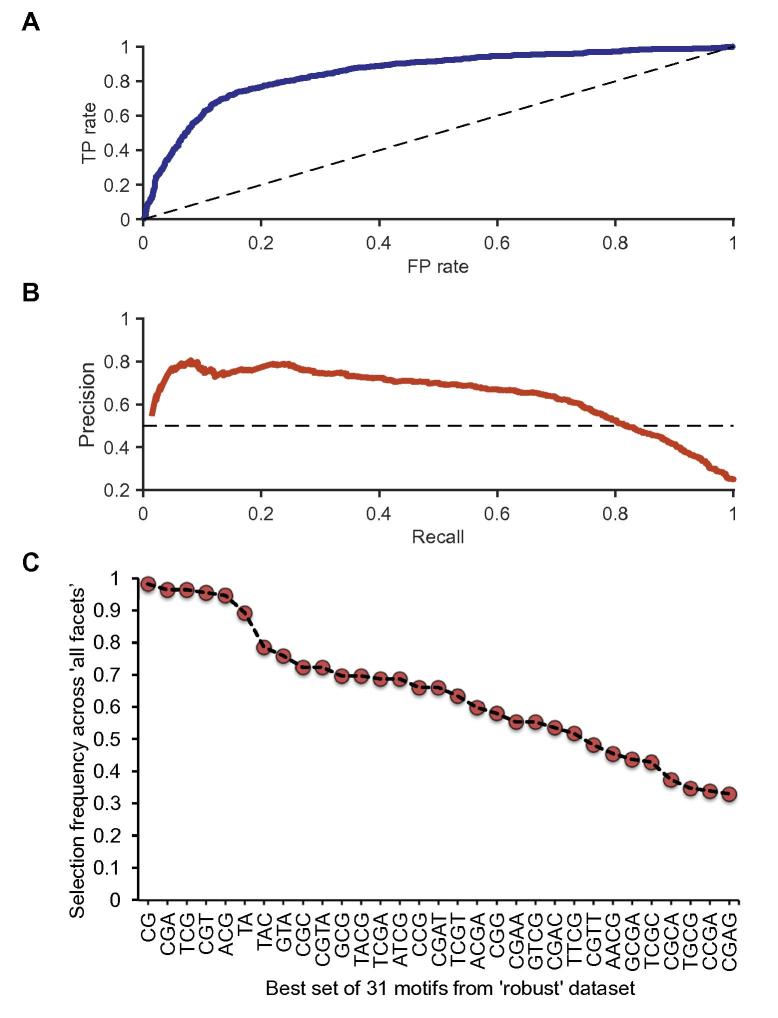
**Classification performance of TELS on the FANTOM5 ‘robust-set’ of TrEns** **A.** ROC curve for discriminating TrEns using the set of 31 motifs identified by TELS; **B.** PR curve for discriminating TrEns using the set of 31 motifs identified by TELS; **C.** Selection frequencies of the 31 selected motifs. The 31 motifs used for discriminating the ‘robust set’ are shown on the X axis, whereas the y-axis shows the selection frequency (as the percentage of the datasets where the respective motif is selected) of the corresponding motifs presented on the X axis. This frequency is calculated across all FANTOM5 cell types/tissues from the ‘all-facets’ dataset from Figure 2. ROC, receiver operating characteristic; PR, precision–recall.

Next, we compare the results obtained using the ‘robust set’ of TrEns with the results achieved by analyzing the ‘all facets’ dataset (Figure S5). In particular, by aggregating the motif signatures per cell type/tissue from the ‘all facets’ dataset, we observe that specific motifs are selected with high frequency across 112 cell types/tissues. We then plotted the set of 31 motifs obtained from the ‘robust set’ and considered as ‘best’ according to the selection frequency of every individual motif across 112 cell types/tissues from the ‘all facets’ dataset. As a result, we observed that six out of the 31 motifs are selected more than 80% of times across different cell types/tissues. Figure 3 shows the ROC and PR curves, respectively, obtained using the combinations of 31 motifs (Figure 3A and B), as well as their corresponding selection frequency of these motifs (Figure 3C). Overall, using this motif set we report an average AUROC of 0.854 ± 0.009 and AUPRC of 0.67 ± 0.01 across 300 experiments.

Notably, some motifs, namely CG, CGA, TCG, CGT, ACG, and TA, almost always help maximizing discrimination performance (Figure 3C). We also observed that two di-nucleotides, CG and TA, are very frequent in the context of other identified kmers. Interestingly, this observation has been explored experimentally in *Drosophila* [20] and reported by another independent study [5]. Among the aforementioned 31 motifs, 10 are tri-nucleotides rich in CG and 19 are tetra-nucleotides also rich in CG. Different from our approach, Colbran et al. [22] used different sequence characteristics coupled with support vector machines (SVM) model to identify informative sequence patterns that distinguish ‘broadly active enhancers’ from random or ‘context-specific’ background. Note that the ‘broadly active enhancers’ analyzed in [22] covered 1961 FANTOM5 enhancer sequences selected based on their expression levels. The sequence patterns we identified by TELS were derived from the complete FANTOM5 ‘robust set’ of TrEns that contains 38,554 enhancer sequences.

### Analyzing TrEns expressed only in single FANTOM5 cell types or tissues

The hypothesis we investigate in this subsection is whether or not FANTOM5 enhancers expressed and transcribed exclusively in one cell type/tissue can be distinguished based on their sequence characteristics, from TrEns expressed exclusively in different cell types/tissues. To explore this hypothesis, we applied TELS to identify motif signatures that can discriminate effectively the TrEns of the FANTOM5 ‘exclusively transcribed’ dataset, from those of the corresponding ‘negative exclusively transcribed’ datasets. Due to the insufficient number of training samples, 16 out of 112 cell types/tissues were excluded from the analysis. The classification performance achieved across the remaining 96 cell types/tissues is presented in Figures S7 and S8. Our results show, that ‘exclusively transcribed’ enhancers can be distinguished from ‘negative exclusively transcribed’ set with an average PPV and GM of 65.23% ± 0.87 and 65.02% ± 0.68, respectively, with PPV >80% in some cell types/tissues (∼25 cases). However, PPV is about 60% in ∼40 cell types/tissues, indicating that in addition to the identified motif signatures, other factors have strong influence on cell type/tissue-specific TrEn activation.

### Performance comparison with existing approaches using FANTOM5 data

In this subsection we assess TELS performance over existing approaches. To do so, we compare the discriminative capabilities of the motif set identified by TELS, with motif sets reported by other studies. In this way, one could assess how good are the motifs selected by TELS. These other motif sets include (1) a set of 20 informative 6-mers that were used by linear SVM to distinguish chromatin-defined enhancers from random DNA sequences [21]; (2) motifs CA and GA, as well as the AP-1 binding site motif, being among the most discriminative for enhancer activation as derived from self-transcribing active regulatory region sequencing (STARR-seq) experiments in Drosophila [20]; and (3) a set of 351 sequence characteristics used as input to a complex ensemble model of 1000 SVMs in dragon ensemble enhancer predictor (DEEP) for prediction of both transcribed and chromatin-defined enhancers on a genome-wide scale [37].

Comparing motif signatures identified by different computational approaches is not straightforward for several reasons. First, the considered computational methods are trained and tested on different datasets. For example, Lee et al. used enhancers defined by ChIP-seq [21], whereas Yanez-Cuna et al. used a quantitative experimental approach to measure enhancers’ activity in Drosophila [20]. Second, there are differences in the selection of machine learning models and tuning of model parameters (*e.g.*, C parameter for SVM or number of SVMs in the ensemble).

Since it is not feasible to re-train all models included in the comparison on FANTOM5 data, we used the reported motifs from [20], [21], [37], and tested the classification performances using FANTOM5 data. To make the comparison more fair, we focus on two classifiers, the K-nearest neighbor (KNN) and bagged decision trees (BDT), not used in our study or by the methods we compare with, for training models and selecting features. Thus, our evaluation provides a more objective picture of the generalization capabilities of different motif sets. KNN and BDT are implemented in Matlab and optimized using different sets of parameters, namely, the values for K were selected to be 3, 4, 5, 6, 7, 8, or 20 for KNN, while the values for B were selected to be 20, 30, 40, 50, 60, 70, or 150 for BDT. The best set of parameters in terms of the GM classification performance was selected based on the results of the fine-tuning experimentation for KNN and BDT (*i.e.*, 8 neighbors provide better results for KNN and 150 trees for BDT for all methods) (Figure S9). To assess the classification performance for all sets of motifs, we repeat the training and testing process 100 times using the best set of parameters. In every individual run we split the data (*i.e.*, enhancers and non-enhancers) randomly into training (60%) and testing (40%) sets. Please note that we used here different splitting of training and testing sets for performance assessment (compared to the 20% training and 80% testing we used before for motif identification).

**Figure 4:**
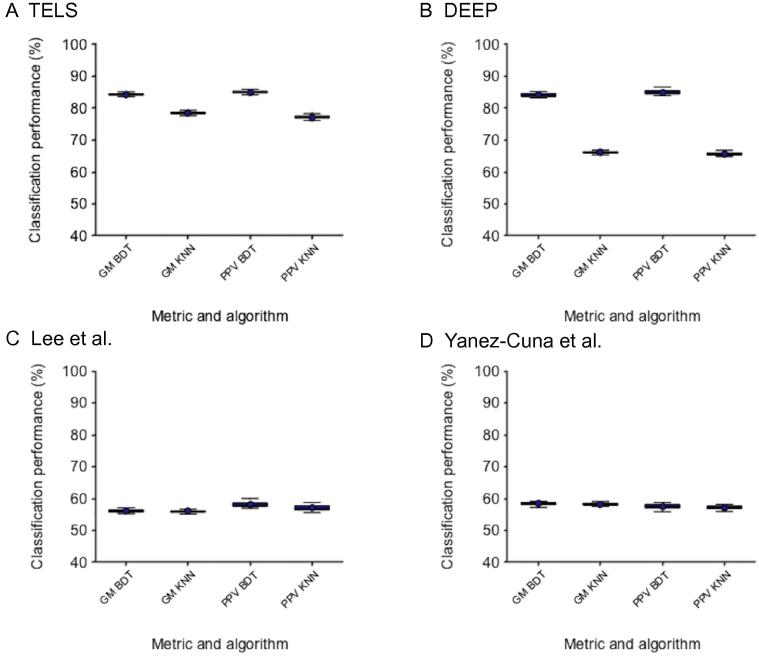
**Classification performance of motif sets identified by different methods on FANTOM5 TrEns** Shown in the plots is the classification performance using motif signatures identified by TELS (**A**), DEEP [37] (**B**), Lee et al. [21] (**C**), and Yanez-Cuna et al. [20] (**D**), respectively. For each method, the classification performance (in %) in terms of GM and PPV was evaluated using two classification algorithms BDT and KNN. BDT, bagged decision trees; KNN, k-nearest neighbors; PPV, positive predictive value; GM, geometric mean of sensitivity and specificity.

As shown in Figure 4, the set of 31 motifs identified by TELS discriminates much more accurately the ‘robust set’ of TrEn compared to motif sets used by other studies. Since the differences in the performance between TELS and DEEP using the BDT classifier appear marginal, we applied the Vargha and Delaney statistical test to quantify practically those small differences in performance [38]. TELS always appears to perform better than DEEP with GM 84.34% ± 0.32 and PPV 85.05% ± 0.37. The superiority of TELS in terms of performance is consistent using two different classification algorithms. In fact, the results presented here indicate that the motif signatures reported by TELS are very effective in recognition of FANTOM5 enhancers defined by CAGE experiments. It should be noted that, the major advantage of TELS over DEEP is the great model simplification (*i.e.*, DEEP is a complex ensemble model). The number of features used by TELS is 31, while the number of features used by DEEP is 351 and thus ∼11.3 times larger.

From the technical point of view, TELS achieves comparable classification performance using three independent classification methods namely LR, BDT, and KNN. This indicates that our findings are not biased to one particular classification model, although we used LR during the motif selection. Moreover, our results indicate that the classification algorithm used for assessing the motifs’ importance results in no bias in the motif selection process.

### Performance comparison with existing approaches using ENCODE data

To assess more thoroughly TELS performance on independent data, we then test the discriminative capabilities of the motif signatures identified by TELS on chromatin-defined enhancers reported by ENCODE [24]. With the ENCODE enhancer datasets, we also evaluate the discrimination capabilities of the sets of motifs identified by DEEP [37], Lee et al. [21], and Yanez-Cuna et al. [20]. This comparison analysis provides important insights into the robustness of the developed framework and the generalization capabilities of the identified motifs using completely independent classifiers tested on unseen data.

To do so, we utilize all chromatin-defined ENCODE enhancers that do not overlap with the CAGE-defined enhancers from the FANTOM5 atlas [5]. As input variables, TELS was tested using the set of 31 motif signatures derived from the ‘robust set’ of TrEns, whereas DEEP, Lee et al*.*, and Yanez-Cuna et al. methods were tested using their original motif signatures. For classification, we used KNN and BDT algorithms under the best setting of parameters based on our fine-tuning experimentation. To assess the classification performance, we measure the GM in 100 runs, where in each run we split the data randomly into training (60%) and testing (40%) sets.

**Figure 5:**
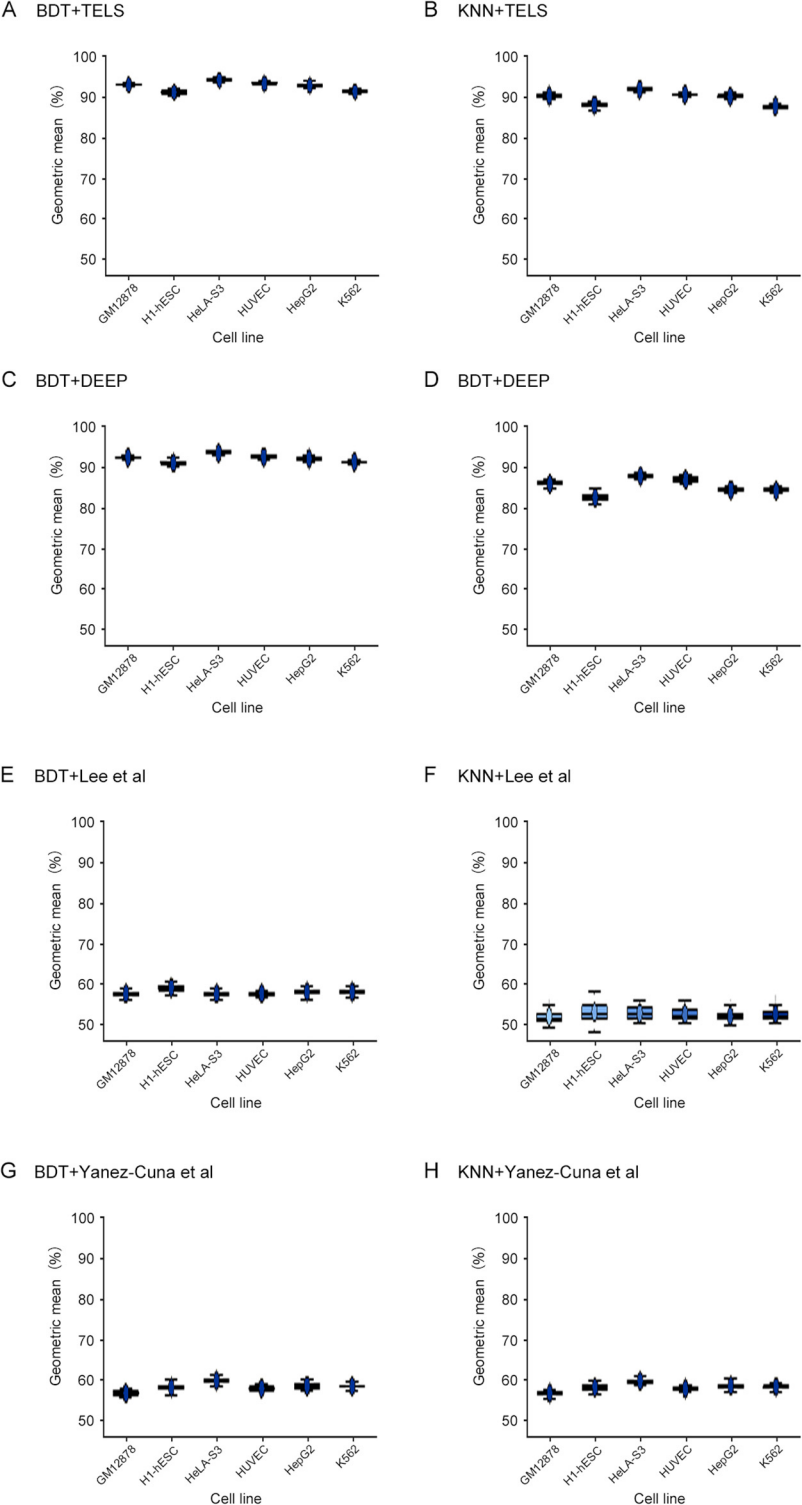
**Classification performance of motif sets identified by different methods on chromatin-defined enhancers obtained from ENCODE** The classification performance (%) presented in the plots is measured in terms of GM across different cell lines from ENCODE as shown in the x-axis. Classification performance using motifs identified by TELS was evaluated using two classification algorithms BDT (**A**) and KNN (**B**), respectively. Classification performance using motifs identified by DEEP [37] was evaluated using two classification algorithms BDT (**C**) and KNN (**D**), respectively. Classification performance using motifs identified by Lee et al. [21] was evaluated using two classification algorithms BDT (**E**) and KNN (**F**), respectively. Classification performance using motifs identified by Yanez-Cuna et al. [20] was evaluated using two classification algorithms BDT (**G**) and KNN (**H**), respectively. The classification performance (%) presented in the y-axis is measured in terms of GM across different cell-lines from ENCODE as shown in the x-axis. ENCODE, Encyclopedia of DNA Elements; hESC, human embryonic stem cell; HUVEC, human umbilical vein endothelial cell.

Our results demonstrate that the set of 31 motif signatures identified by TELS is more effective than the motif signatures identified by other methods when tested on the set of chromatin-defined enhancers from ENCODE (Figure 5). Our findings also indicate that TELS reveals DNA sequence characteristic of TrEns that are common to chromatin-defined enhancers, and thus similar sequence motifs are equally predictive of chromatin-defined (ENCODE) and of transcribed (FANTOM5) enhancers. Biologically, our findings might also indicate that many of the ‘strong’ enhancers defined by ChIP-seq are transcribed and/or that there are common DNA sequence characteristics for all poised and active enhancers [5], [15]. More importantly, the results using ENOCDE data re-confirm that TELS can be used to decipher effectively motif signatures of enhancers compared to existing approaches.

## Conclusion

In this study, we developed TELS, a novel machine learning framework for identifying predictive motif signatures of TrEns. First we applied TELS to CAGE-defined enhancers from FANTOM5. This allows us to compile a comprehensive catalog of motif signatures from different cell types/tissues. The use of reported motif signatures as presented in our study results in models with improved capability of discrimination of TrEns in comparison with models that use other existing motif sets determined for the same purpose. In addition, our study is the first one to report combinations of motifs that maximize classification performance of TrEns that are exclusively transcribed in one cell type/tissue from those that are exclusively transcribed in all other cell types/tissues. Moreover, by analyzing the so-called ‘robust set’ of TrEns, our study identified 31 frequently selected motifs predictive of TrEn broad activity. As an additional validation step, we show that the TELS-identified motif signatures can also discriminate with high classification performance chromatin-defined enhancers from different ENCODE datasets. Consequently, our analysis reports combinations of motifs that allow us to discriminate TrEns and chromatin-defined enhancers more effectively, compared to the motif sets reported using other methods.

Nonetheless, the proposed bioinformatics method allows for many future improvements. For instance, performing the same analysis on TrEn data obtained by single cell analysis, if available by FANTOM, will eliminate potential biases caused by cell population heterogeneity and may lead to more fine-grained results about the enhancer genomic landscape. In addition, applying the same analysis to CAGE-defined promoters from FANTOM5 will answer equally important questions about promoters’ sequence characteristics ‘encrypted' within their genomic sequence. Lastly, we would like to point out that stratifying TrEn data by their expression levels similarly to the data reported by Arner et al. [9] and our laboratory [10], and inferring the expression levels of TrEns using sequence characteristics, may complement the findings presented in this study.

## Authors’ contributions

DK and VBB conceived the project, analyzed the data, and wrote the manuscript. DK and HA performed the experiments. All authors read and approved the final manuscript.

## Competing interests

The authors have declared no competing interests.

## Supplementary material

Supplementary File S1 **TELS additional implementation details**

Supplementary Figure S1 **Classification performance using alternative filtering feature selection methods** Shown in the plots is the classification performance in terms of PPV (%) using optimized set of motifs selected by mRMR feature selection (**A**) and Fisher’s exact test (**B**) for all cell types/tissues from the ‘all-facets’ dataset, respectively. PPV, positive predictive value; mRMR, minimum redundancy and maximum relevancy.

Supplementary Figure S2 **TELS classification performance in terms of GM and PPV for discriminating 112 cell types/tissues from the FANTOM5 ‘all-facets’ dataset versus ‘all facets random controls’ dataset** Classification performance in terms of GM (%) and PPV (%) using the combination of 31 motifs for 112 cell types/tissues from FANTOM5 ‘all-facets’ dataset.

Supplementary Figure S3 **TELS classification performance in terms of AUROC and AUPRC for discriminating 112 cell types and tissues from the FANTOM5 ‘all-facets’ dataset versus ‘all facets random controls’ dataset** The AUROC and AUPRC for discriminating the ‘all-facet’ dataset from negative controls using the combination of 31 motifs are presented in **A** and **B**, respectively. AUROC, area under the receiver operating characteristic curve; AUPRC, area under the precision recall.

Supplementary Figure S4 **TELS classification performance using the set of best motifs across 49 FANTOM5 cell types/tissues from ‘all-facets’ dataset A.** ROC curves for discriminating TrEns from ‘all-facets’ dataset from negative controls. **B.** PR curves for discriminating TrEns from ‘all-facets’ dataset from negative controls. In all ROC curves, the diagonal lines in black correspond to the classification performance of a random predictor. Only 49 out of 112 cell types/tissues from the all-facets are shown here due to the space limitation. The full set of ROC and PR curves across all cell types/tissues is available at http://www.cbrc.kaust.edu.sa/TELS/. TP, true positive; FP, false positive.

Supplementary Figure S5 **The atlas of the most informative motifs across 112 cell types/tissues from the ‘all facets’ dataset** Different cell types/tissues from FANTOM5 (112 in total) are presented on the X axis, whereas the considered motifs are shown on the Y axis grouped by the length of the motifs as di-nucleotide motifs (**A**), tri-nucleotide motifs (**B**), and tetra-nucleotide motifs (**C**−**E**). In all panels, the informative motifs available in the respective cell types/tissues are shown in blue, whereas motifs that were not selected in the respective cell types/tissues were left blank.

Supplementary Figure S6 **Analysis of motif signatures and DNA sequences of TrEns that belong to tissues of different developmental stages A.** Similarity matrix based on the Jaccard index constructed from the best tissue-specific motif sets for nine randomly selected tissues that belong to different developmental stages; **B.** Similarity matrix based on the Jaccard index constructed from the actual input enhancer sequences of the nine tissues from panel A.

Supplementary Figure S7 **TELS classification performance on TrEns expressed in only one cell type/tissue versus all other ‘exclusively transcribed’ datasets** Classification performance indicated by GM (%) and PPV (%) using the combination of 31 motifs for 96 cell types/tissues from FANTOM5 ‘exclusively transcribed’ datasets.

Supplementary Figure S8 **TELS classification performance in terms of PPV (%) across 96 cell types/tissues from the ‘exclusively transcribed’ datasets** We show the corresponding number of motifs that maximize MCC (*i.e.*, called overall ‘best’ motifs) selected by TELS across 96 cell types and tissues (X axis), versus the corresponding PPV (Y axis). In total 16 out of 112 FANTOM5 cell types/tissues were excluded from analyses due to insufficient number of training samples.

Supplementary Figure S9 **Fine tuning of classification parameters (GM) for two algorithms used in the comparative analysis A.** Optimizing the number of decision trees for BDT; **B.** Optimizing the number of nearest neighbors for KNN.
