# Supplementary material for: TELS: A Novel Computational Framework for Identifying Motif Signatures of Transcribed Enhancers
Source: Genomics Proteomics Bioinformatics. 2018 Dec 19;16(5):332–41. doi: 10.1016/j.gpb.2018.05.003 (PMC6364045; doi:10.1016/j.gpb.2018.05.003)
Supplement: Supplementary File S1 — TELS additional implementation details [file mmc1.docx]

**File S1 TELS additional implementation details**

To achieve more robust results and provide a more comprehensive view of the recognition performance, we repeat the learning process for every classification problem 300 times, and compute the average classification performance for every combination of top-ranked features. This process guarantees stable selection of combinations of features since it is based on the average classification performance of multiple runs that involve random splits of the input data. In summary, we generate the following classification problems. (1) “All facets” vs. “all-facets random controls” comparison involves 112 cell types/tissues. For each cell type/tissue we evaluate 346 combinations of motifs using 300 random splits of the data. (2) For “Robust” set vs. “robust random controls” comparison, we evaluate 346 combinations of motifs using 300 random splits of the data. (3) “Exclusively transcribed” vs. “exclusively transcribed negative controls” comparison involves 96 cell types/tissues. For each cell type/tissue, we evaluate 346 combinations of motifs using 300 random splits of the data.

**Classification performance metrics**

To assess the classification performance and identify combinations of motifs that minimize classification error, we consider the following performance metrics.

(1) (1)

where and .

(2)

, (3)

where TP, FP, FN, TN, GM, PPV, and MCC denote true positives, false positives, false negatives, true negatives, geometric mean of sensitivity and specificity, positive predictive value, and Mathews correlation coefficient, respectively.

**Gini-based feature selection**

Feature selection based on the Gini-index is frequently found in the literature as ‘Gini importance’ or simply Gini FS. Gini-index can be utilized to indicate the relative importance of individual features of the feature vector [26,34]. In other words, Gini-index provides a score that corresponds to the relative ranking of the features, and is considered a by-product of the random forest classifier training. Technically speaking, at each node *b* of the binary tree *B* in the forest, the optimal split is sought using the Gini impurity denoted as *i(b)*. Gini impurity is an approximation to entropy, which measures how well a split separates the samples of the two classes in the considered node. The Gini impurity for node *b* is calculated using the formula below

*i(b)* = *1 - p_1_^2^ – p_0_^2^*  (4)

where p_k_ = n_k_/n is the fraction of the *n_k_* samples from class *k* = {0,1} out of the total number of samples at node *b*.

We also define decrease *Di(b)* as shown below.

*Di(b)* = i(b) – pl(bl) – pr(br) (5)

where pl and pr are the sample fractions by splitting nodes *bl* and *br* for one variable named *θ* of the vector. Considering all variables in the feature vector, the optimal split $Di\theta(b,B)$ can be determined.

Finally, we define the Gini-importance (IG) based on the formula below.

(6)

This index indicates how often a particular feature *θ* was selected for a split, and quantifies its discriminative power for the classification problem of interest.

**Comparison of Gini-index to alternative FS methods**

We compare the recognition performance of LR with Gini index using two other state-of-the-art algorithms for FS, namely minimum redundancy maximum relevance criterion (mRMR) and Fisher’s test-based FS. To conduct a fair comparison, we follow the same protocols summarized in **Figure 1**. For all competitor methods, we repeat the feature ranking 300 times using a random subset of the data samples equal to 20%. From them, we select the most frequent ranking as the best. We would like to point out here, that the feature ranking is a completely independent process that does not involve any interaction with classifier or other algorithm.

After applying individual feature ranking, we use greedy selection method and estimate the classification performance using all top-*N* ranked feature subsets (*N* =1, 2, 3, …, 346). We repeat the learning process 300 times, and we select the top-ranked subset that achieves the maximum MCC. All implementations are made in Matlab R2014b using the FEAST library for FS. Results obtained by mRMR and the Fisher’s test with LR are summarized in Figure S1 for all cell type/tissue-specific enhancers included in the ‘all-facets’ dataset. FS using a combination of LR with Gini index achieves higher recognition performance in almost all of the tested cases. In particular, FS based on Gini index achieves an average PPV of 85.94% and MCC of 0.72 across all studied tissues and cell types. On the other hand, FS by mRMR achieves average PPV of 84.05% and MCC of 0.67, whereas FS by Fisher’s test achieves average PPV of 85.13% and MCC of 0.71, respectively. This clearly suggests that FS that combines LR with Gini index under the greedy forward selection is the best choice.

**Analysis of motif signatures across tissues that belong to different developmental stages**

We use nine tissues that belong to three different developmental stages, namely ectoderm (brain, spinal cord, and eye), mesoderm (kidney, heart, and spleen), and endoderm (lung, liver, and pancreas) according to the Embryonic Development & Stem Cell Compendium (<https://discovery.lifemapsc.com/in-vivo-development>).

For the selected tissues, we generate the pairwise similarity matrix using the Jaccard index (*i.e.*, considering exact matches) for the sets of motif signatures (Figure S6). We also generate the pairwise similarity matrix using the Jaccard index (*i.e.*, considering exact matches) for the corresponding sets of input TrEn sequences (Figure S6B). Our results show that different sets of TrEns are ‘weakly’ similar to each other based on their enhancer sequences (Figure S6B). Tissues that belong to ectoderm, mesoderm, and endoderm have average pairwise similarity of 0.129, 0.056, and 0.207, respectively, whereas pancreas and liver appear the most similar tissues based on their enhancer sequences. As expected, tissues that are more similar in terms of enhancer sequences have in a way more similar sets of motif signatures. The average pairwise Jaccard similarity across all nine sets of motif signatures is 0.022. Mesoderm tissues have completely disjoint sets of motifs (*i.e.*, the numbers of selected motifs are only 8, 8, and 4 for kidney, heart, and spleen, respectively). Apparently, heart tissue appears similar to lung and pancreas that belong to a different developmental stage, both in terms of enhancer sequences and selected motifs.
