# Supplementary material for: TELS: A Novel Computational Framework for Identifying Motif Signatures of Transcribed Enhancers
Source: Genomics Proteomics Bioinformatics. 2018 Dec 19;16(5):332–41. doi: 10.1016/j.gpb.2018.05.003 (PMC6364045; doi:10.1016/j.gpb.2018.05.003)
Supplement: Supplementary Figure S1 — Classification performance using alternative filtering feature selection methods Shown in the plots is the classification performance in terms of PPV (%) using optimized set of motifs selected by mRMR feature selection (A) and Fisher’s exact test (B) for all cell types/tissues from the ‘all-facets’ dataset, respectively. PPV, positive predictive value; mRMR, minimum redundancy and maximum relevancy. [file mmc2.pptx]

## Slide 1
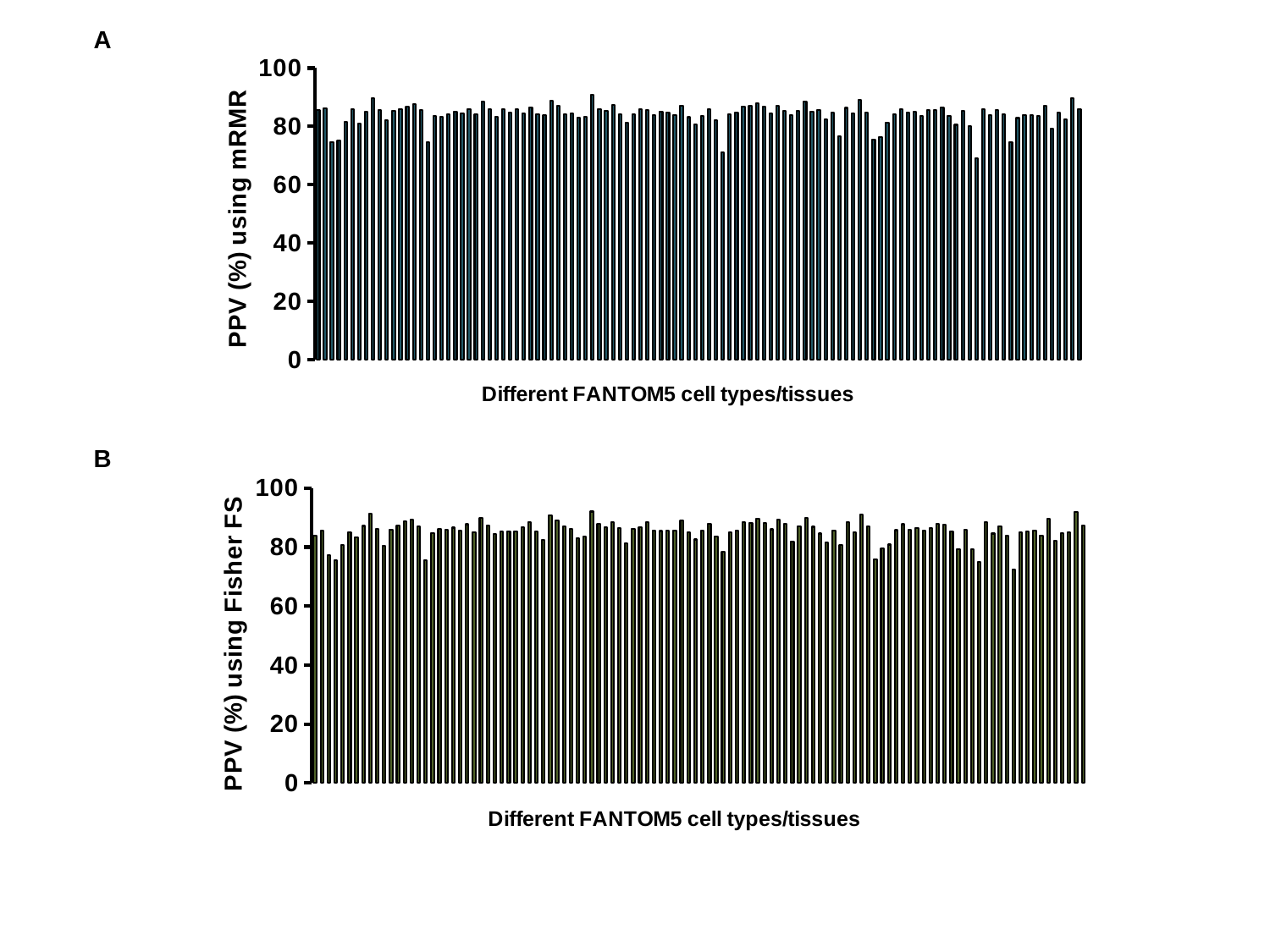

### Chart
| Category | |
|---|---|
| epithelial cell of esophagus | 85.70968873075218 |
| eye | 86.2846253965675 |
| penis | 74.65690784913548 |
| skin of body | 75.22980122352618 |
| hepatocyte | 81.48237524759926 |
| melanocyte | 85.76481153132 |
| fibroblast of the conjuctiva | 81.0705223990322 |
| fat cell | 85.01196562412525 |
| granulocyte | 89.70197151114178 |
| amniotic epithelial cell | 85.61870525245523 |
| lymph node | 82.11677632075506 |
| endothelial cell of hepatic sinusoid | 85.43836879685541 |
| astrocyte | 85.92798483845252 |
| blood vessel endothelial cell | 86.68185361174572 |
| macrophage | 87.51350435252552 |
| brain | 85.46133767068598 |
| parotid gland | 74.44473421997012 |
| esophagus | 83.6706531033772 |
| acinar cell | 83.18481250984 |
| ciliated epithelial cell | 84.18079814373013 |
| blood vessel | 85.1046996520116 |
| throat | 84.4300579232118 |
| cardiac fibroblast | 85.91787048494209 |
| small intestine | 84.12941204563121 |
| blood | 88.39304886784748 |
| fibroblast of tunica adventitia of artery | 85.76433498387603 |
| trabecular meshwork cell | 83.35888835055474 |
| spinal cord | 85.77692797379142 |
| chondrocyte | 84.58867456999958 |
| bronchial smooth muscle cell | 85.8936927214129 |
| hair follicle cell | 84.43312492415141 |
| spleen | 86.4235527662401 |
| placenta | 84.2596300810529 |
| corneal epithelial cell | 83.79039770951108 |
| dendritic cell | 88.80307682588445 |
| skin fibroblast | 86.92988485942915 |
| epithelial cell of Malassez | 84.07841858952031 |
| epithelial cell of prostate | 84.41218826037363 |
| reticulocyte | 82.98307101228643 |
| cardiac myocyte | 83.31950338741252 |
| basophil | 90.70050829717428 |
| fibroblast of gingiva | 85.7554425468192 |
| enteric smooth muscle cell | 85.25285013360536 |
| circulating cell | 87.44403288640058 |
| fibroblast of lymphatic vessel | 84.19244409558127 |
| iris pigment epithelial cell | 81.15765328117683 |
| fibroblast of choroid plexus | 84.17938858749838 |
| placental epithelial cell | 86.00626601594293 |
| gingival epithelial cell | 85.6832217859534 |
| fibroblast of pulmonary artery | 83.9206374238161 |
| endothelial cell of lymphatic vessel | 85.00851643631958 |
| heart | 84.67341784699468 |
| kidney | 83.87039630198684 |
| T cell | 87.15707586054738 |
| internal male genitalia | 83.13370986620451 |
| testis | 80.70106159658545 |
| hepatic stellate cell | 83.6030176860301 |
| myoblast | 86.01261801062948 |
| skeletal muscle tissue | 82.08009449514122 |
| submandibular gland | 71.11157023445011 |
| liver | 84.13864991081388 |
| thyroid gland | 84.68644482330141 |
| lung | 86.86579908578918 |
| kidney epithelial cell | 87.17465455155912 |
| mesenchymal cell | 87.80059783321738 |
| lymphocyte of B lineage | 86.6965934591846 |
| mammary epithelial cell | 84.55892515722111 |
| mast cell | 87.18843549512631 |
| meninx | 85.3999432548005 |
| smooth muscle cell of the esophagus | 83.82715743779798 |
| mesothelial cell | 85.2964629501763 |
| natural killer cell | 88.492954532486 |
| keratinocyte | 85.02582552002568 |
| female gonad | 85.57274429427068 |
| salivary gland | 82.28378414402002 |
| retinal pigment epithelial cell | 84.74961147777383 |
| neuronal stem cell | 76.69435000659936 |
| respiratory epithelial cell | 86.32356746044658 |
| lens epithelial cell | 84.41877488637128 |
| neutrophil | 89.12927354667558 |
| osteoblast | 84.59308780818351 |
| pancreas | 75.4647646734256 |
| olfactory region | 76.39996199675912 |
| keratocyte | 81.1793964618425 |
| pericyte cell | 84.1879026681653 |
| preadipocyte | 85.8770650429971 |
| smooth muscle cell of prostate | 84.7113859629935 |
| adipose tissue | 85.06974177714568 |
| prostate gland | 83.55570499893568 |
| tonsil | 85.72224971114721 |
| sensory epithelial cell | 85.5537655988773 |
| skeletal muscle cell | 86.41146912097065 |
| uterine smooth muscle cell | 83.63030650742313 |
| smooth muscle cell of trachea | 80.50755474971045 |
| large intestine | 85.37771173378043 |
| smooth muscle tissue | 80.18320114544197 |
| stomach | 69.04201718697608 |
| stromal cell | 85.9153895313192 |
| tendon cell | 83.8976790335405 |
| thymus | 85.61487014859107 |
| tongue | 84.01860731358153 |
| umbilical cord | 74.44699024565521 |
| uterus | 82.83451962960613 |
| urinary bladder | 83.72590622151938 |
| urothelial cell | 83.9758236065758 |
| vagina | 83.53820496584675 |
| vascular associated smooth muscle cell | 87.17904727115346 |
| neuron | 79.21573134546311 |
| intestinal epithelial cell | 84.60377319096722 |
| gallbladder | 82.36463231013312 |
| monocyte | 89.5670908857661 |
| fibroblast of periodontium | 85.78168613985788 |A
B
### Chart
| Category | |
|---|---|
| epithelial cell of esophagus | 83.7743412299001 |
| eye | 85.46099248936585 |
| penis | 77.3873141046724 |
| skin of body | 75.42291496836901 |
| hepatocyte | 80.777045707007 |
| melanocyte | 85.02876153156721 |
| fibroblast of the conjuctiva | 83.4048621383334 |
| fat cell | 87.14943245463425 |
| granulocyte | 91.18079605149266 |
| amniotic epithelial cell | 86.12253461670807 |
| lymph node | 80.31102514672878 |
| endothelial cell of hepatic sinusoid | 85.9129182772363 |
| astrocyte | 87.367247304209 |
| blood vessel endothelial cell | 88.73434805722185 |
| macrophage | 89.25390180443281 |
| brain | 87.13519087785104 |
| parotid gland | 75.45881334684698 |
| esophagus | 84.7889237923823 |
| acinar cell | 86.03809695636912 |
| ciliated epithelial cell | 85.77432640878078 |
| blood vessel | 86.57693654228538 |
| throat | 85.61140205470402 |
| cardiac fibroblast | 87.72356269420791 |
| small intestine | 84.96500070385838 |
| blood | 89.94441034097873 |
| fibroblast of tunica adventitia of artery | 87.25505321896628 |
| trabecular meshwork cell | 84.53683887045068 |
| spinal cord | 85.16469255396997 |
| chondrocyte | 85.16307493235176 |
| bronchial smooth muscle cell | 85.40708546500356 |
| hair follicle cell | 86.66213663716218 |
| spleen | 88.4656019873244 |
| placenta | 85.39681202942631 |
| corneal epithelial cell | 82.46588719722271 |
| dendritic cell | 90.82871257496257 |
| skin fibroblast | 88.9271897130526 |
| epithelial cell of Malassez | 86.9262758262403 |
| epithelial cell of prostate | 86.26610463365168 |
| reticulocyte | 82.90876517979432 |
| cardiac myocyte | 83.5952138549914 |
| basophil | 92.03267753006521 |
| fibroblast of gingiva | 87.9505533244823 |
| enteric smooth muscle cell | 86.73129328399575 |
| circulating cell | 88.46128793977982 |
| fibroblast of lymphatic vessel | 86.53696022889763 |
| iris pigment epithelial cell | 81.36847503685573 |
| fibroblast of choroid plexus | 86.07069126478778 |
| placental epithelial cell | 86.63622048409982 |
| gingival epithelial cell | 88.43735868462743 |
| fibroblast of pulmonary artery | 85.5378712894952 |
| endothelial cell of lymphatic vessel | 85.42389839337392 |
| heart | 85.63122697376468 |
| kidney | 85.5414987994385 |
| T cell | 89.1039177817797 |
| internal male genitalia | 85.12190777204447 |
| testis | 82.57072725067437 |
| hepatic stellate cell | 85.56327439263632 |
| myoblast | 87.76055268096256 |
| skeletal muscle tissue | 83.55918081465506 |
| submandibular gland | 78.45416034179073 |
| liver | 84.9052750396108 |
| thyroid gland | 85.51553418373395 |
| lung | 88.48571065619412 |
| kidney epithelial cell | 88.2051020053313 |
| mesenchymal cell | 89.65833525066633 |
| lymphocyte of B lineage | 88.2104664705329 |
| mammary epithelial cell | 86.0035602342854 |
| mast cell | 89.31767887157038 |
| meninx | 87.82922850774568 |
| smooth muscle cell of the esophagus | 81.77868862779525 |
| mesothelial cell | 86.91109781876975 |
| natural killer cell | 89.81191847593736 |
| keratinocyte | 86.87070851742205 |
| female gonad | 84.5624405057782 |
| salivary gland | 81.49528848164081 |
| retinal pigment epithelial cell | 85.57263253274795 |
| neuronal stem cell | 80.634045666328 |
| respiratory epithelial cell | 88.49275283783811 |
| lens epithelial cell | 85.08919192709318 |
| neutrophil | 91.00178230745662 |
| osteoblast | 86.90825720131981 |
| pancreas | 75.77904858829578 |
| olfactory region | 79.59073575702126 |
| keratocyte | 80.83124341295168 |
| pericyte cell | 85.7134319916984 |
| preadipocyte | 87.73432886795008 |
| smooth muscle cell of prostate | 85.72828988774968 |
| adipose tissue | 86.4870981874724 |
| prostate gland | 85.5504135439325 |
| tonsil | 86.3733826157351 |
| sensory epithelial cell | 87.80820944915726 |
| skeletal muscle cell | 87.601760619863 |
| uterine smooth muscle cell | 85.17825746050141 |
| smooth muscle cell of trachea | 79.4017546395918 |
| large intestine | 85.83787285461473 |
| smooth muscle tissue | 79.25964111049443 |
| stomach | 74.8519764571475 |
| stromal cell | 88.40868459904021 |
| tendon cell | 84.5673646336501 |
| thymus | 86.92515272307388 |
| tongue | 83.75945721284258 |
| umbilical cord | 72.25042462494005 |
| uterus | 85.10755493452038 |
| urinary bladder | 85.31289528369278 |
| urothelial cell | 85.67366744941688 |
| vagina | 83.96102804921316 |
| vascular associated smooth muscle cell | 89.47458277470598 |
| neuron | 82.06580487057602 |
| intestinal epithelial cell | 84.72724152521631 |
| gallbladder | 85.08188255918591 |
| monocyte | 91.86504895541032 |
| fibroblast of periodontium | 87.1972031242806 |
