# Supplementary figures and images for: TELS: A Novel Computational Framework for Identifying Motif Signatures of Transcribed Enhancers
Source: Genomics Proteomics Bioinformatics. 2018 Dec 19;16(5):332–41. doi: 10.1016/j.gpb.2018.05.003 (PMC6364045; doi:10.1016/j.gpb.2018.05.003)

## Slide 1
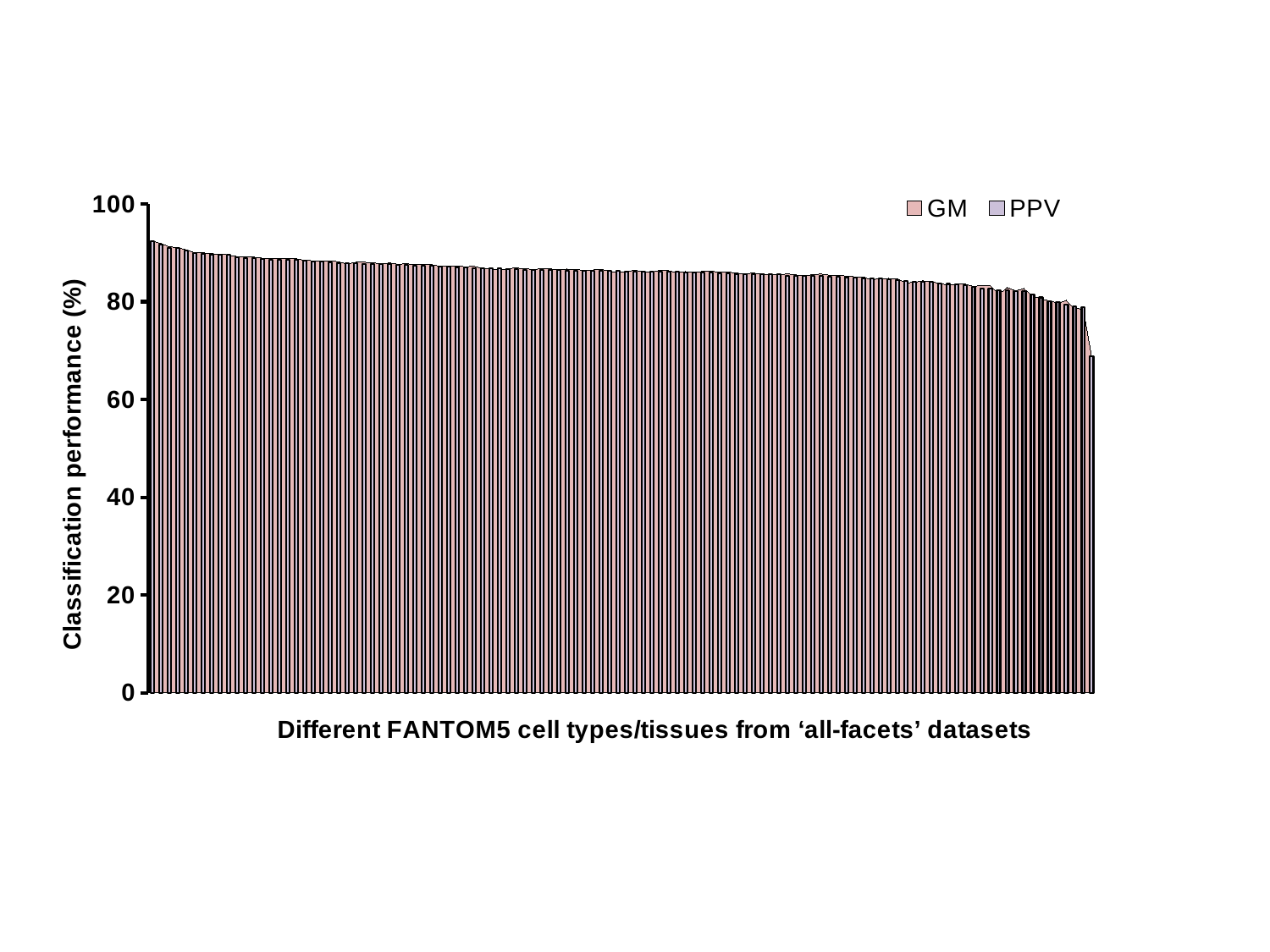

### Chart
| Category | | |
|---|---|---|

Supplement: Supplementary Figure S2 — TELS classification performance in terms of GM and PPV for discriminating 112 cell types/tissues from the FANTOM5 ‘all-facets’ dataset versus ‘all facets random controls’ dataset Classification performance in terms of GM (%) and PPV (%) using the combination of 31 motifs for 112 cell types/tissues from FANTOM5 ‘all-facets’ dataset. [file mmc3.pptx]

## Slide 1
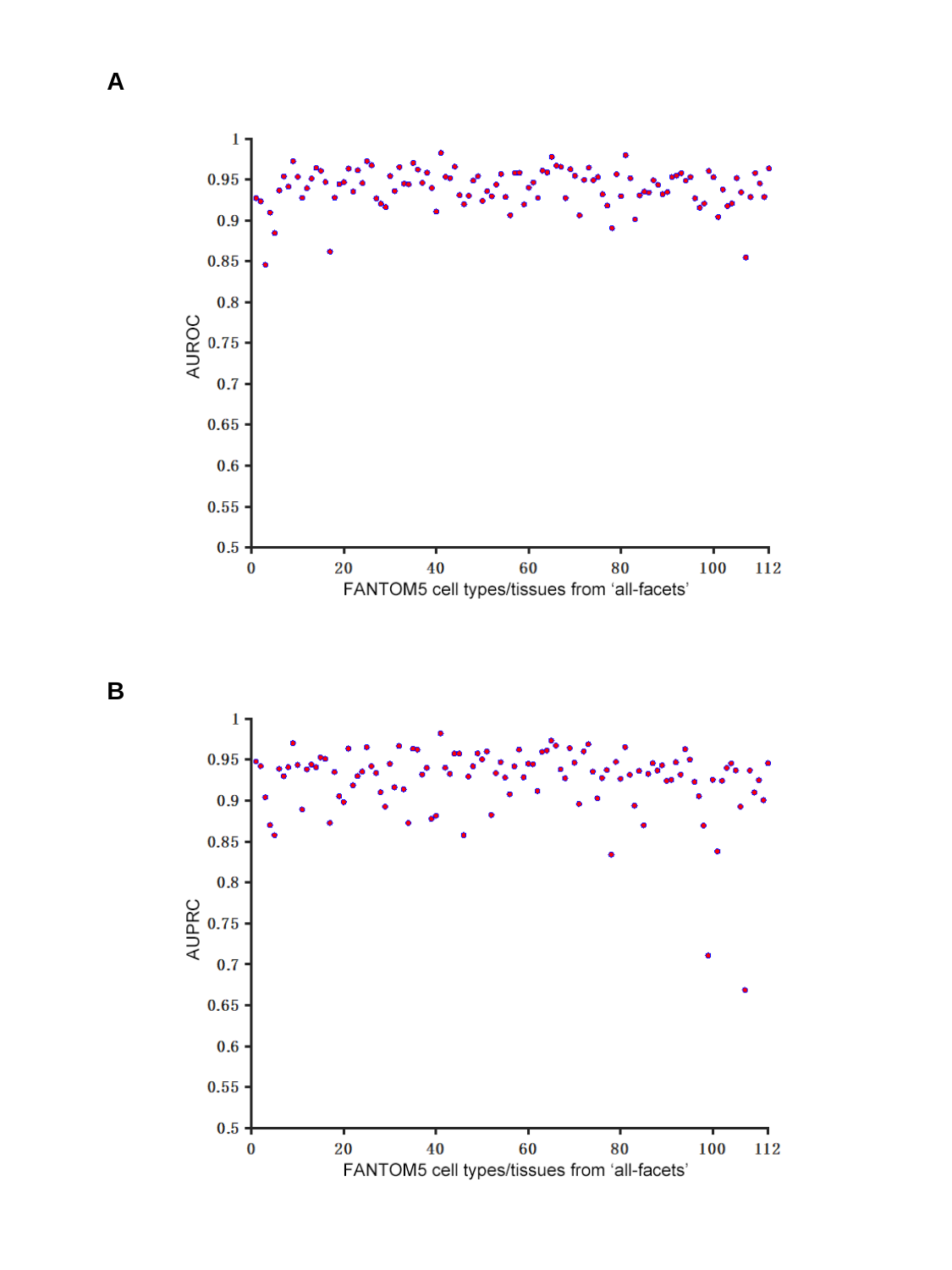

A
B

Supplement: Supplementary Figure S3 — TELS classification performance in terms of AUROC and AUPRC for discriminating 112 cell types and tissues from the FANTOM5 ‘all-facets’ dataset versus ‘all facets random controls’ dataset The AUROC and AUPRC for discriminating the ‘all-facet’ dataset from negative controls using the combination of 31 motifs are presented in A and B, respectively. AUROC, area under the receiver operating characteristic curve; AUPRC, area under the precision recall. [file mmc4.pptx]

## Slide 1
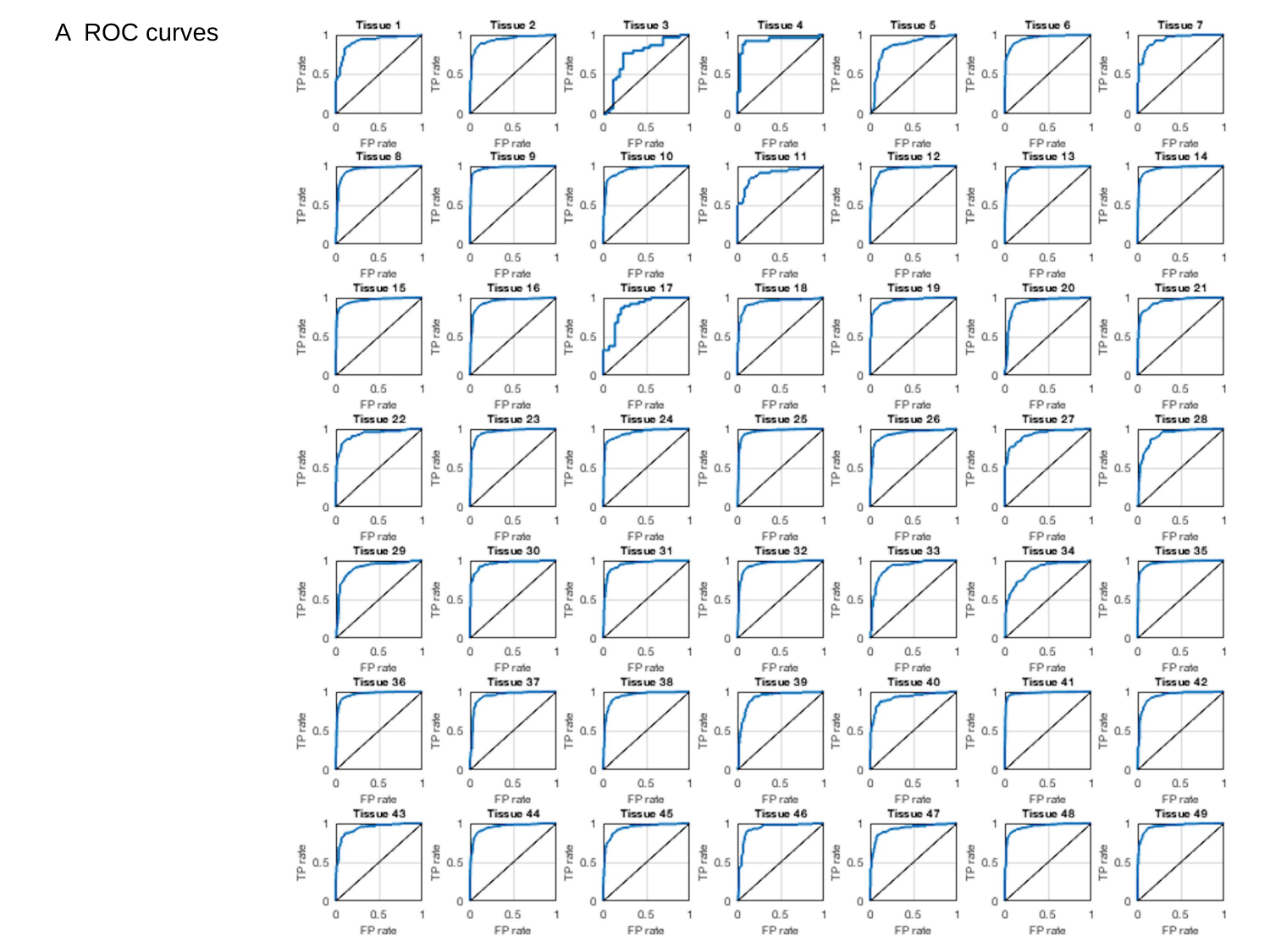

A ROC curves

## Slide 2
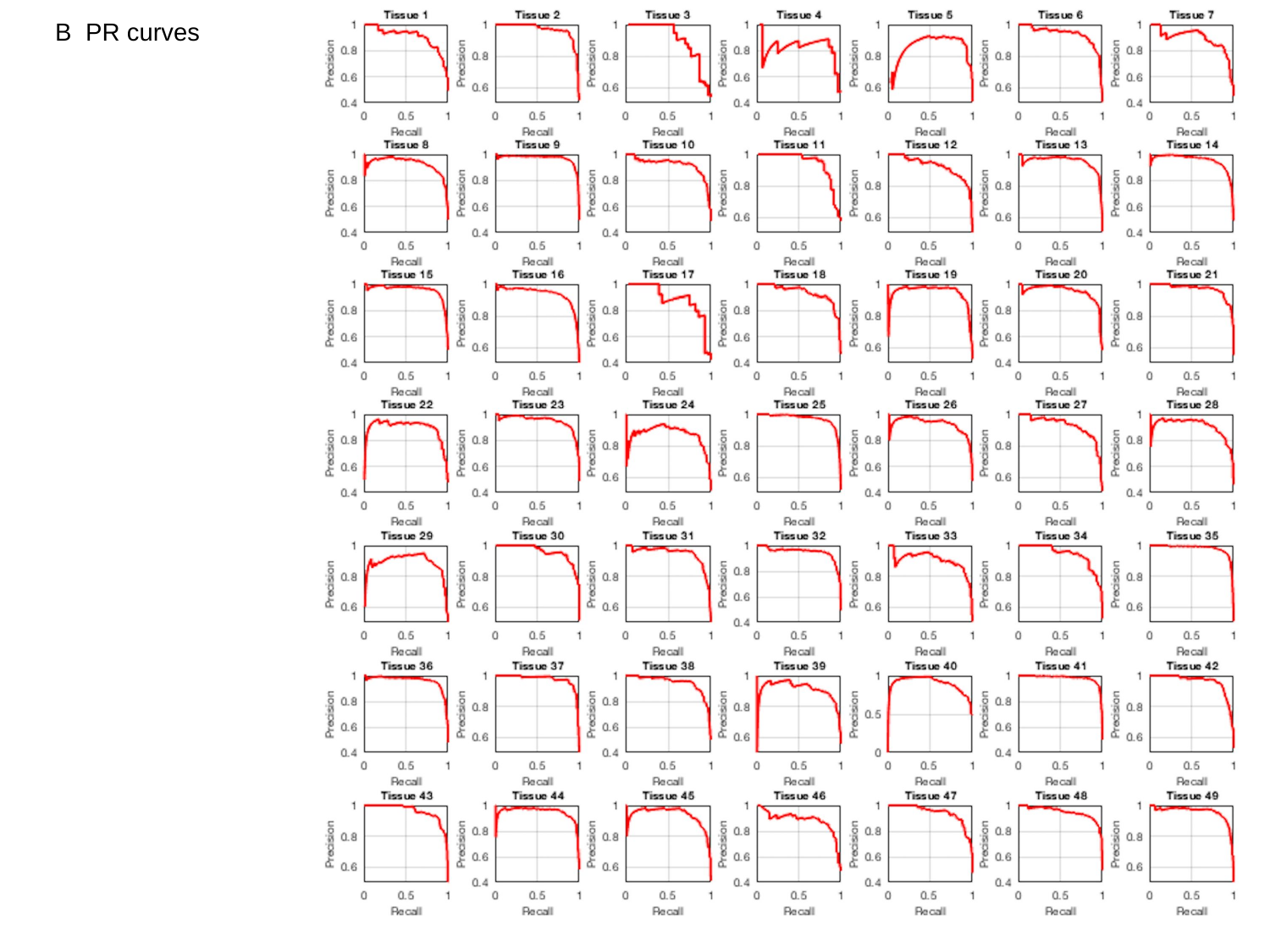

B PR curves

Supplement: Supplementary Figure S4 — TELS classification performance using the set of best motifs across 49 FANTOM5 cell types/tissues from ‘all-facets’ dataset A. ROC curves for discriminating TrEns from ‘all-facets’ dataset from negative controls. B. PR curves for discriminating TrEns from ‘all-facets’ dataset from negative controls. In all ROC curves, the diagonal lines in black correspond to the classification performance of a random predictor. Only 49 out of 112 cell types/tissues from the all-facets are shown here due to the space limitation. The full set of ROC and PR curves across all cell types/tissues is available at http://www.cbrc.kaust.edu.sa/TELS/. TP, true positive; FP, false positive. [file mmc5.pptx]

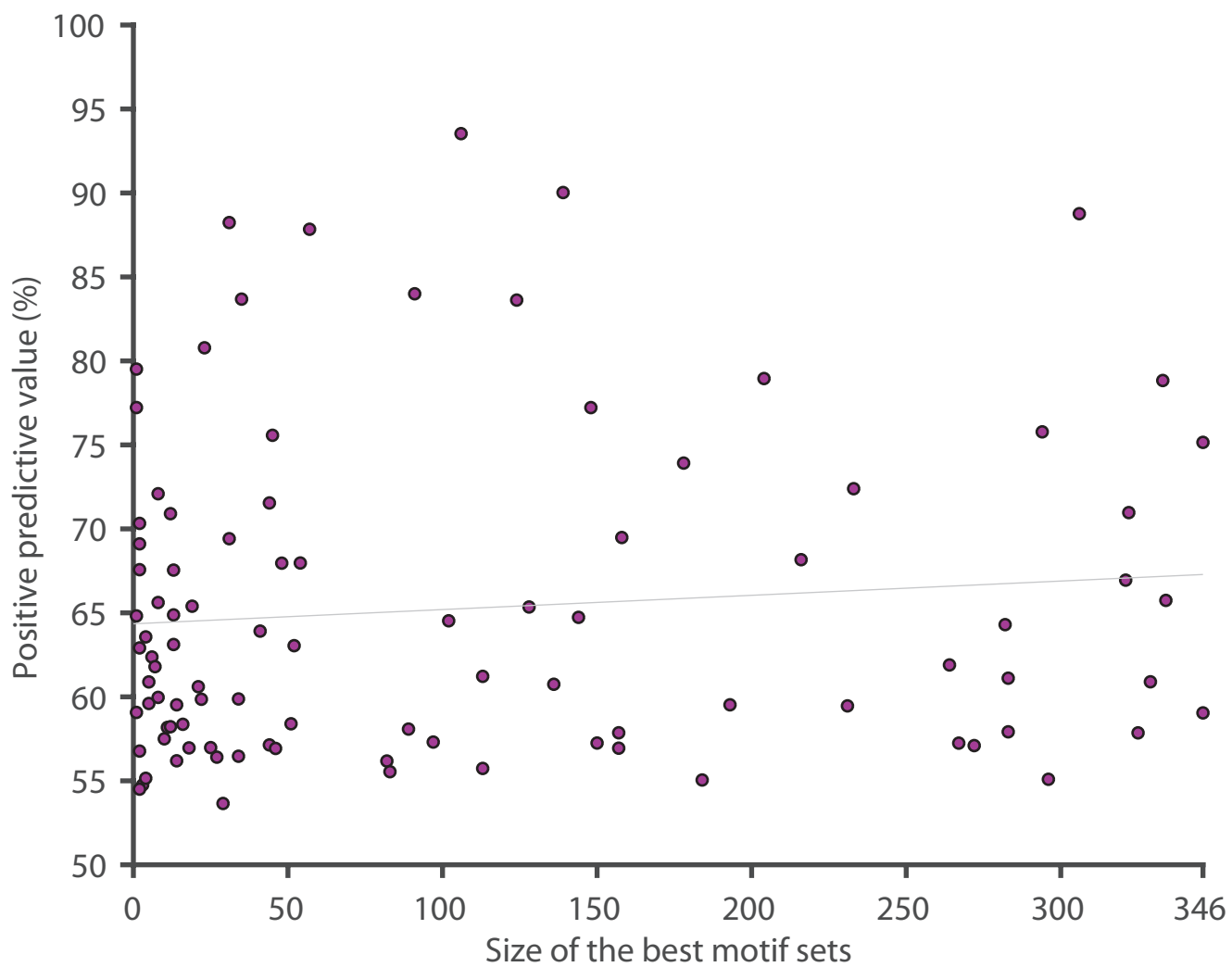

Supplement: Supplementary Figure S8 — TELS classification performance in terms of PPV (%) across 96 cell types/tissues from the ‘exclusively transcribed’ datasets We show the corresponding number of motifs that maximize MCC (i.e., called overall ‘best’ motifs) selected by TELS across 96 cell types and tissues (X axis), versus the corresponding PPV (Y axis). In total 16 out of 112 FANTOM5 cell types/tissues were excluded from analyses due to insufficient number of training samples. [file mmc9.pdf]

## Slide 1
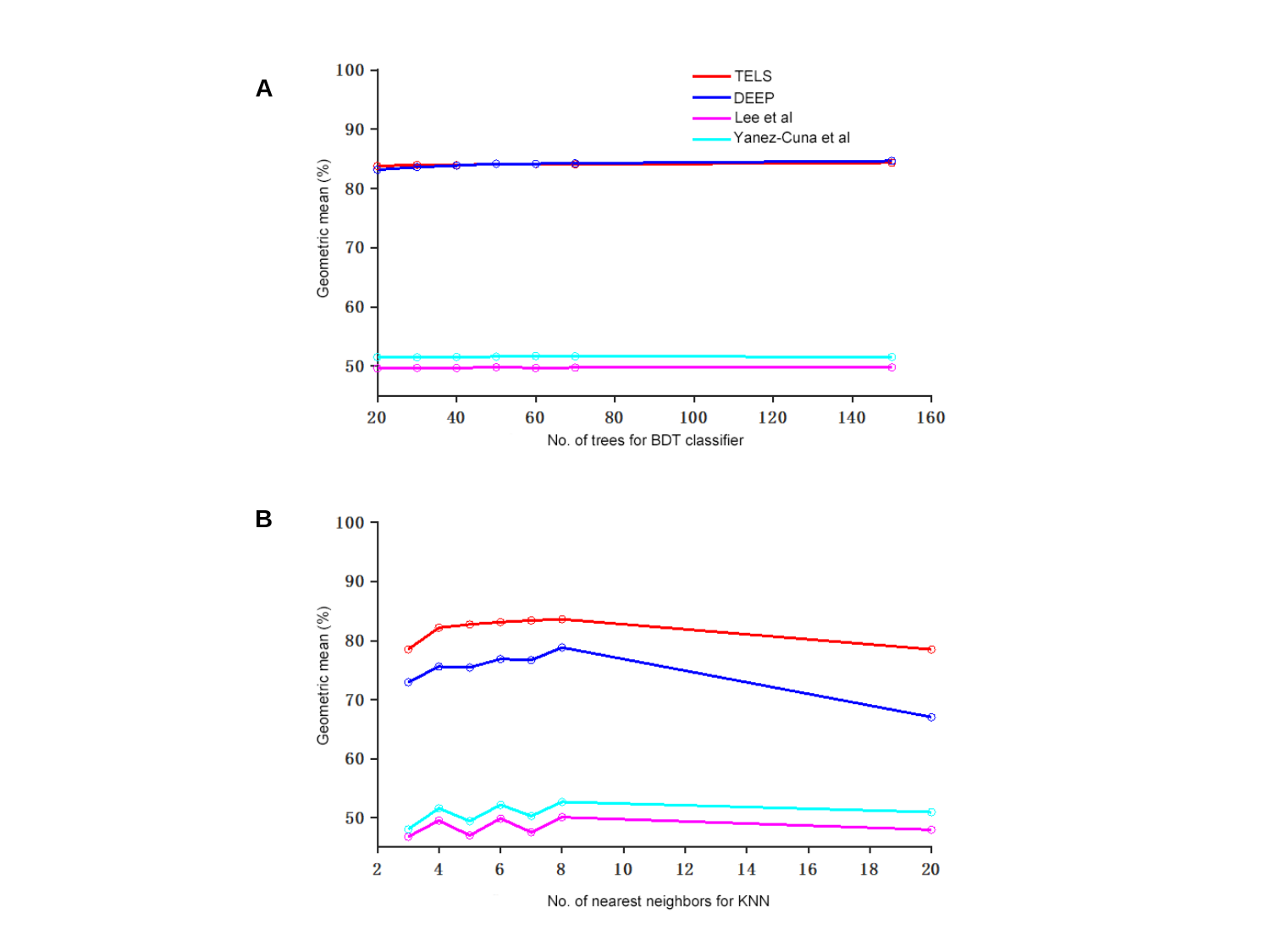

A
B

Supplement: Supplementary Figure S9 — Fine tuning of classification parameters (GM) for two algorithms used in the comparative analysis A. Optimizing the number of decision trees for BDT; B. Optimizing the number of nearest neighbors for KNN. [file mmc10.pptx]
