# Supplementary material for: TELS: A Novel Computational Framework for Identifying Motif Signatures of Transcribed Enhancers
Source: Genomics Proteomics Bioinformatics. 2018 Dec 19;16(5):332–41. doi: 10.1016/j.gpb.2018.05.003 (PMC6364045; doi:10.1016/j.gpb.2018.05.003)
Supplement: Supplementary Figure S5 — The atlas of the most informative motifs across 112 cell types/tissues from the ‘all facets’ dataset Different cell types/tissues from FANTOM5 (112 in total) are presented on the X axis, whereas the considered motifs are shown on the Y axis grouped by the length of the motifs as di-nucleotide motifs (A), tri-nucleotide motifs (B), and tetra-nucleotide motifs (C−E). In all panels, the informative motifs available in the respective cell types/tissues are shown in blue, whereas motifs that were not selected in the respective cell types/tissues were left blank. [file mmc6.pptx]

## Slide 1
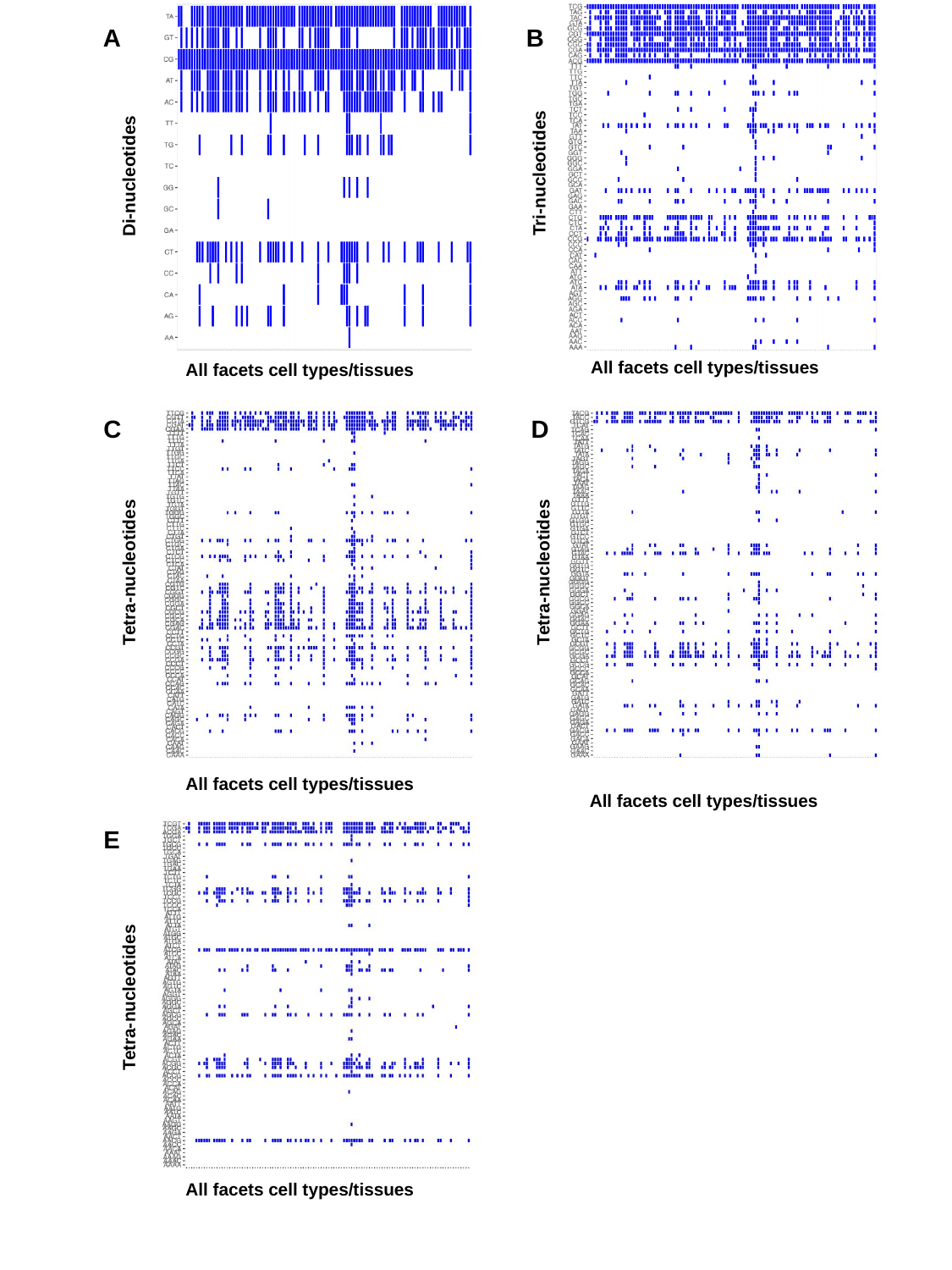

A
A
A
A
B
Tri-nucleotides
Di-nucleotides
All facets cell types/tissues
All facets cell types/tissues
C
D
Tetra-nucleotides
Tetra-nucleotides
All facets cell types/tissues
All facets cell types/tissues
E
Tetra-nucleotides
All facets cell types/tissues
