# Supplementary material for: TELS: A Novel Computational Framework for Identifying Motif Signatures of Transcribed Enhancers
Source: Genomics Proteomics Bioinformatics. 2018 Dec 19;16(5):332–41. doi: 10.1016/j.gpb.2018.05.003 (PMC6364045; doi:10.1016/j.gpb.2018.05.003)
Supplement: Supplementary Figure S6 — Analysis of motif signatures and DNA sequences of TrEns that belong to tissues of different developmental stages A. Similarity matrix based on the Jaccard index constructed from the best tissue-specific motif sets for nine randomly selected tissues that belong to different developmental stages; B. Similarity matrix based on the Jaccard index constructed from the actual input enhancer sequences of the nine tissues from panel A. [file mmc7.pptx]

## Slide 1
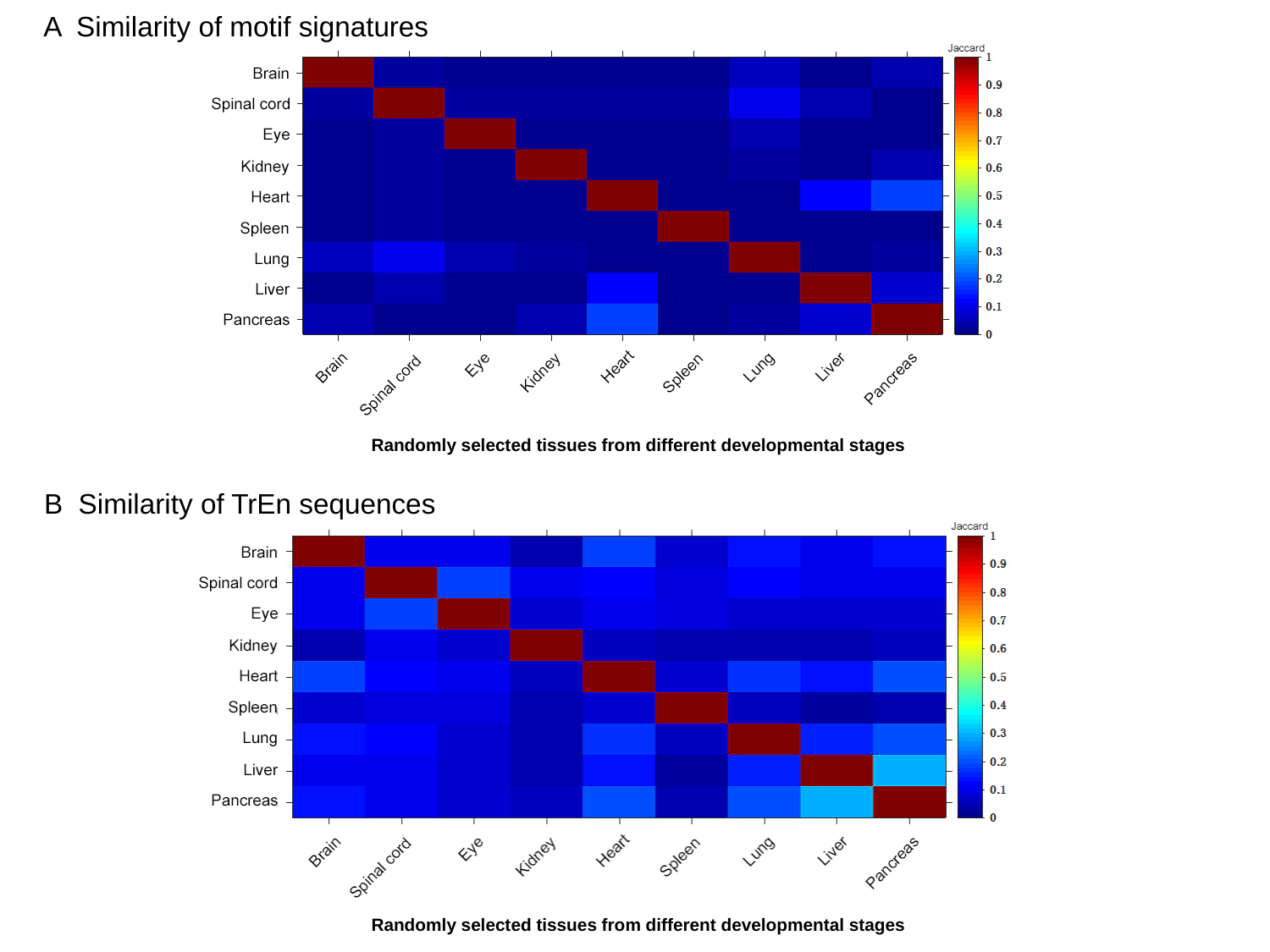

A Similarity of motif signatures
Randomly selected tissues from different developmental stages
B Similarity of TrEn sequences
Randomly selected tissues from different developmental stages
