# Supplementary material for: TELS: A Novel Computational Framework for Identifying Motif Signatures of Transcribed Enhancers
Source: Genomics Proteomics Bioinformatics. 2018 Dec 19;16(5):332–41. doi: 10.1016/j.gpb.2018.05.003 (PMC6364045; doi:10.1016/j.gpb.2018.05.003)
Supplement: Supplementary Figure S7 — TELS classification performance on TrEns expressed in only one cell type/tissue versus all other ‘exclusively transcribed’ datasets Classification performance indicated by GM (%) and PPV (%) using the combination of 31 motifs for 96 cell types/tissues from FANTOM5 ‘exclusively transcribed’ datasets. [file mmc8.pptx]

## Slide 1
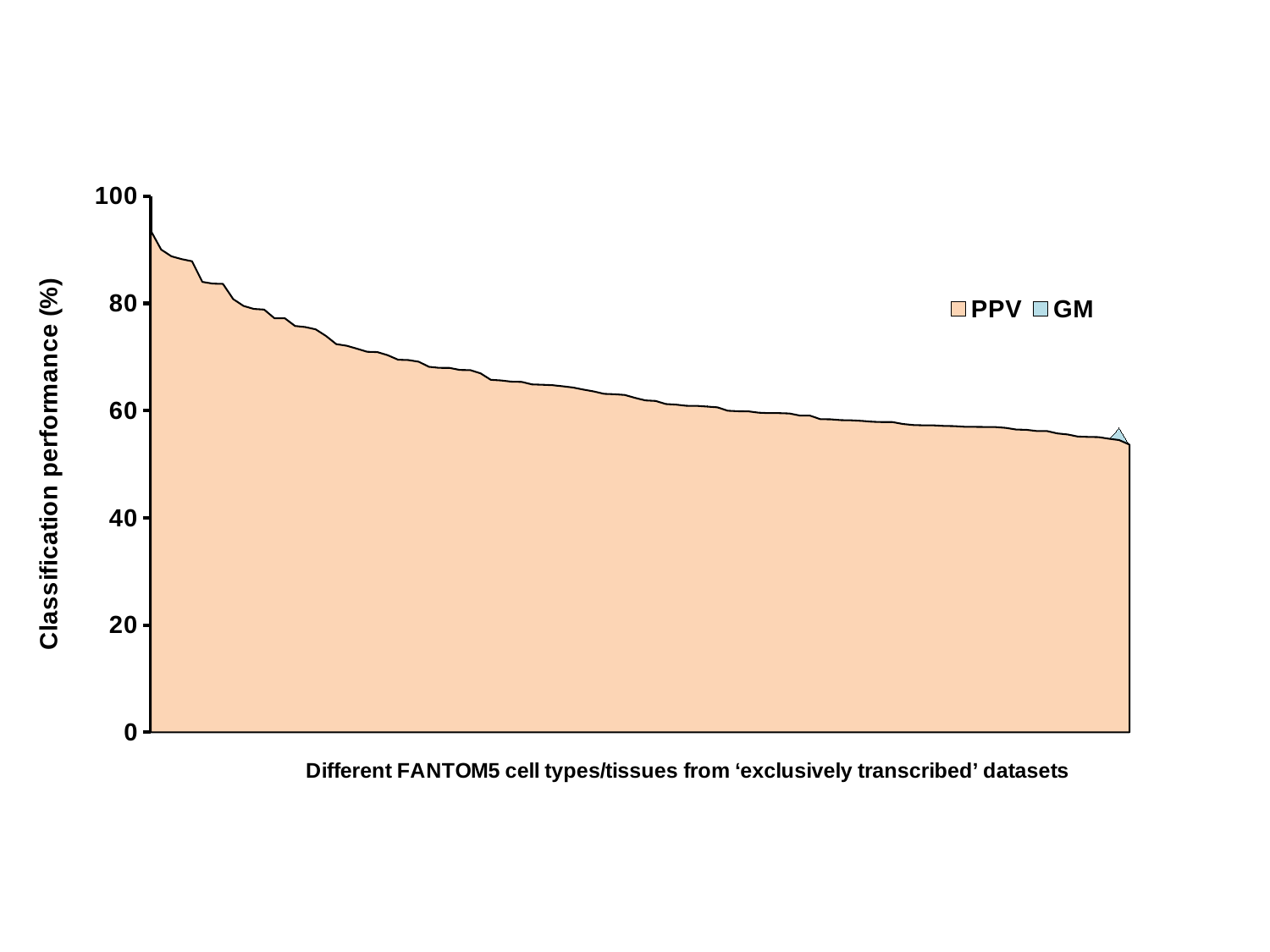

### Chart
| Category | | |
|---|---|---|
| smooth muscle cell of trachea | 93.5321833049106 | 83.9914094291437 |
| smooth muscle tissue | 90.02560163850474 | 79.759321126174 |
| fibroblast of tunica adventitia of artery | 88.7660010240656 | 72.9460666446588 |
| tongue | 88.2380952380952 | 82.44932639386087 |
| stomach | 87.83900226757366 | 81.28120993206312 |
| trabecular meshwork cell | 83.9974937343359 | 75.94176413146776 |
| uterine smooth muscle cell | 83.67891816920935 | 78.00950195954479 |
| vagina | 83.6222838650023 | 71.61581308438267 |
| cardiac myocyte | 80.7823129251701 | 63.88511462281626 |
| parotid gland | 79.5134032634032 | 71.65508168717545 |
| umbilical cord | 78.9509179092512 | 72.4396980201051 |
| fibroblast of choroid plexus | 78.83415195915188 | 73.26492232356908 |
| esophagus | 77.22385141739976 | 60.80490867898035 |
| smooth muscle cell of prostate | 77.22072476789458 | 71.4607546993297 |
| blood vessel | 75.7766990291262 | 66.74554696992136 |
| adipose tissue | 75.5718026631488 | 64.64088200125985 |
| gallbladder | 75.15144772154116 | 68.0905792617721 |
| uterus | 73.91737805289206 | 70.6053789623849 |
| pericyte cell | 72.3925104022192 | 65.88909100656718 |
| heart | 72.09159761683078 | 65.79914005593587 |
| testis | 71.54439175219298 | 70.58273601716142 |
| lens epithelial cell | 70.9698629341153 | 60.33277467323864 |
| neuronal stem cell | 70.90514136448856 | 68.11467847239831 |
| circulating cell | 70.32687006873806 | 70.3891834883652 |
| penis | 69.48623775751436 | 62.612079432447 |
| mast cell | 69.4158047876222 | 68.3165587093469 |
| large intestine | 69.1098614918755 | 60.67337771080093 |
| enteric smooth muscle cell | 68.16559992772589 | 66.17946303180246 |
| fibroblast of gingiva | 67.96886827658506 | 62.28552701106977 |
| endothelial cell of lymphatic vessel | 67.95909512266586 | 66.60200054999585 |
| throat | 67.57149728578298 | 65.90171654487622 |
| natural killer cell | 67.5463635717625 | 66.09208908808952 |
| female gonad | 66.94779116465858 | 63.29493698078313 |
| myoblast | 65.7423604173604 | 59.65102337122178 |
| eye | 65.61582781997416 | 63.40297505360842 |
| pancreas | 65.39573580810246 | 60.56335062651324 |
| ciliated epithelial cell | 65.3492259907594 | 63.06085953234133 |
| T cell | 64.87879720715291 | 63.39088862055868 |
| neuron | 64.81871891871229 | 64.4377209669233 |
| epithelial cell of Malassez | 64.73297913374876 | 62.9303527276879 |
| hair follicle cell | 64.52777219501266 | 62.76990756004168 |
| amniotic epithelial cell | 64.29879570066485 | 58.4844552785053 |
| neutrophil | 63.9141348430807 | 61.76785412477758 |
| spleen | 63.56900977236458 | 61.13144397264274 |
| chondrocyte | 63.11659189861048 | 62.1123399301188 |
| salivary gland | 63.04668461531204 | 60.30031353492318 |
| mesenchymal cell | 62.91305041538367 | 58.1355740790273 |
| fat cell | 62.37194456633897 | 61.62480559953503 |
| tendon cell | 61.8990688196812 | 60.04062226486608 |
| acinar cell | 61.796862589206 | 57.8130026441755 |
| endothelial cell of hepatic sinusoid | 61.2200371321563 | 59.67305566481211 |
| skeletal muscle tissue | 61.1101476021568 | 58.77694625556398 |
| keratinocyte | 60.8961063615638 | 58.31441756635072 |
| mammary epithelial cell | 60.88852335067269 | 58.75890309432715 |
| monocyte | 60.7491467642724 | 60.93745692537088 |
| stromal cell | 60.6035967636591 | 59.77559043053698 |
| kidney | 59.9665910042043 | 59.55821408056244 |
| vascular associated smooth muscle cell | 59.8741596313317 | 59.55698905027549 |
| thymus | 59.85577796369204 | 59.17224522507435 |
| melanocyte | 59.6041715850188 | 57.59135195785016 |
| hepatocyte | 59.5257066019874 | 58.20679217142308 |
| brain | 59.5250961985909 | 59.58518613512973 |
| meninx | 59.4616493789872 | 58.89420094807237 |
| hepatic stellate cell | 59.0727599947751 | 55.24585542267281 |
| small intestine | 59.04398585924007 | 58.24014006077707 |
| cardiac fibroblast | 58.3954592709768 | 57.64871336531566 |
| sensory epithelial cell | 58.3656185251247 | 57.89749176877252 |
| liver | 58.2211641970582 | 57.5532580442011 |
| macrophage | 58.1786877317252 | 56.87380402962962 |
| placenta | 58.08031715663737 | 57.34914104689156 |
| fibroblast of lymphatic vessel | 57.91773863121037 | 58.01485963003637 |
| tonsil | 57.8603969941689 | 57.11288180938141 |
| retinal pigment epithelial cell | 57.85528343973387 | 57.7236411923425 |
| internal male genitalia | 57.4996309274925 | 55.59102849494786 |
| iris pigment epithelial cell | 57.3078749360761 | 55.5127749710748 |
| placental epithelial cell | 57.249745628018 | 53.37124782819212 |
| intestinal epithelial cell | 57.24406387989098 | 57.03899838560794 |
| spinal cord | 57.1368289527265 | 56.462837903389 |
| osteoblast | 57.09749795792748 | 56.20666962596632 |
| dendritic cell | 56.9767938239618 | 56.8008139399691 |
| reticulocyte | 56.9600869053988 | 56.04508728916771 |
| skeletal muscle cell | 56.94347337460708 | 56.73427063627211 |
| epithelial cell of prostate | 56.9281282867964 | 56.80171864558565 |
| gingival epithelial cell | 56.77062457529066 | 54.08266390424404 |
| mesothelial cell | 56.4634423670942 | 55.3484918305691 |
| blood vessel endothelial cell | 56.4094811126397 | 56.05276670316015 |
| lymphocyte of B lineage | 56.191777232767 | 56.10284305666448 |
| lung | 56.1794292632548 | 54.73790564092485 |
| thyroid gland | 55.7374535675397 | 55.53283265087414 |
| preadipocyte | 55.5433305528536 | 54.55790646709941 |
| respiratory epithelial cell | 55.1509507657216 | 54.38405258446043 |
| urothelial cell | 55.0913924050807 | 54.88411841498146 |
| astrocyte | 55.0495199944729 | 54.48810232868056 |
| blood | 54.755947785569 | 54.68976765702129 |
| skin fibroblast | 54.5036342500451 | 56.71846191480945 |
| kidney epithelial cell | 53.65136677443459 | 53.52387261193854 |
